# Stimuli-responsive structural transformation of adaptive peptide nanomaterials toward biomedical applications

**DOI:** 10.1016/j.mtbio.2026.103665

**Published:** 2026-09-10

**Authors:** Jikang Liu, Xunan Kan, Yong Su, Yao Yao, Jiawen Zhao, Jianwei Bao, Yong Jin, Qianli Zou

**Affiliations:** a Research and Industrialization of New Drug Release Technology Joint Laboratory of Anhui Province, School of Pharmacy, Anhui Medical University, Hefei, 230032, China; b Xinjiang Key Laboratory of Natural Medicines Active Components and Drug Release Technology, College of Pharmacy, Xinjiang Medical University, Urumqi, 830017, China

**Keywords:** Nanomaterial, Peptide, Self-assembly, Stimuli-responsive transformation, Drug delivery, Nanotherapeutics

## Abstract

Self-assembling peptide nanomaterials have attracted widespread attention in the biomedical field due to their inherent advantages, such as programmable design, intrinsic bioactivity, and excellent biocompatibility. Especially, peptide nanomaterials can be tailored to possess in vivo microenvironment-responsive morphological characteristics, enabling targeted delivery and enhanced therapy. Herein, this review systematically summarizes in vivo stimuli-responsive morphologically transformable peptide nanomaterials, integrating key biomedical applications, including drug delivery and nanotherapeutics, and emphasizing the structure-activity relationship between morphological transformation and therapeutic efficacy, which is critical for overcoming biological barriers. The responsive design strategies, building blocks, noncovalent interactions, and the responsive nanostructures are discussed. The ability of adaptive morphological changes to overcome multiple biological barriers is highlighted, thereby boosting targeted accumulation, tissue penetration, and therapeutic precision. The challenges and opportunities in developing stimuli-responsive morphologically transformable peptide nanomaterials for biomedical applications are also discussed in depth, providing a deeper insight into the role of smart nanomaterials in improving the efficiency of targeted delivery and therapeutic outcomes.

## Graphical abstract

**Figure:**
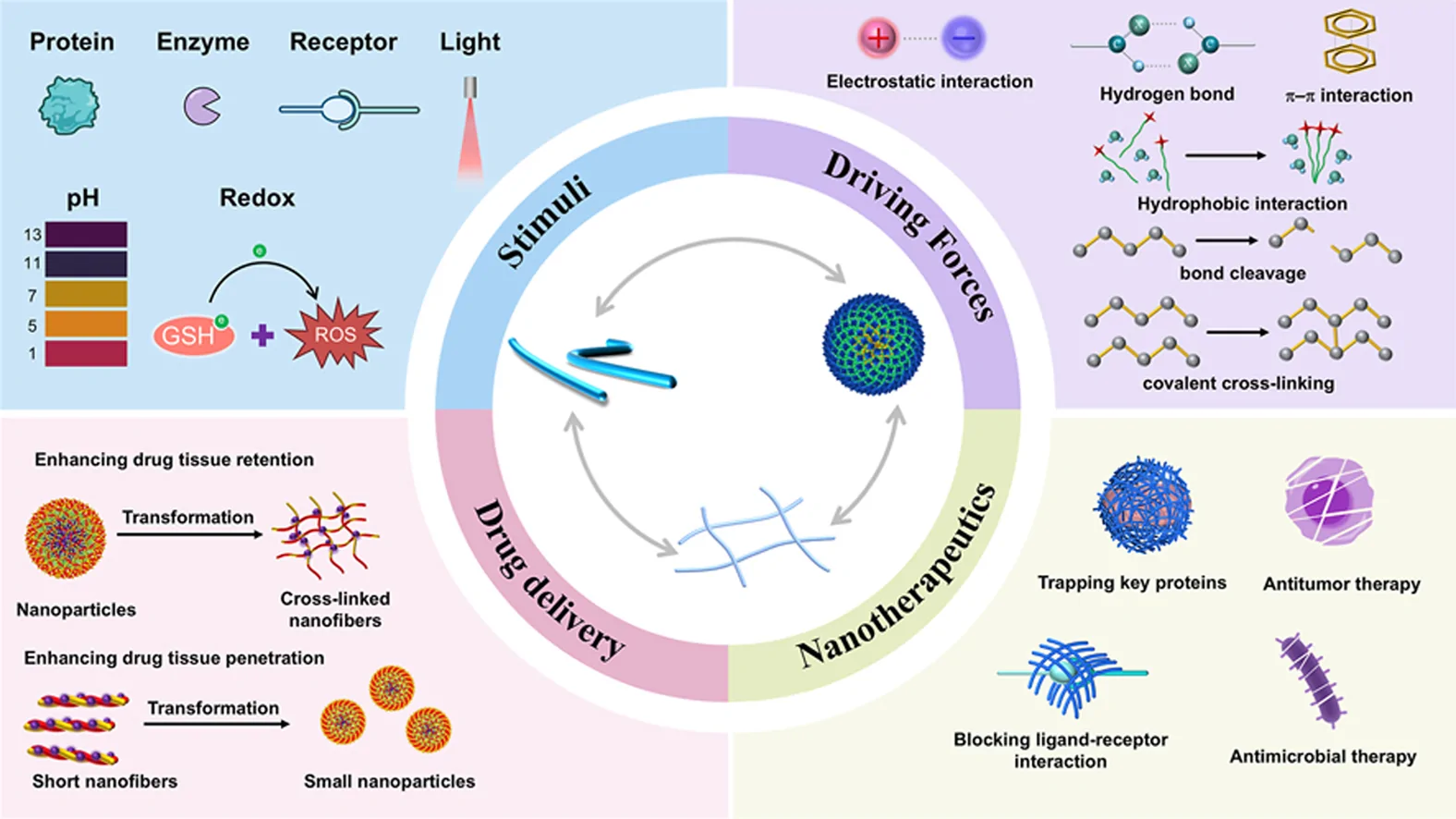

## 1 Introduction

For systemically administered drugs, their therapeutic efficacy is determined not only by pharmaceutical activity but also by the delivery vehicle. The main reason is that without the help of the delivery vehicle, the free drug molecules hardly achieve effective accumulation at the target tissues during blood circulation, resulting in off-target accumulation and toxic side effects. In some cases, the delivery vehicle determines whether potential active pharmaceutical ingredients can ultimately be developed into clinically available drugs [1]. Development of advanced drug delivery systems is important for new medicines development. Additional demands have been placed on drug vehicles, particularly with the introduction of innovative medications incorporating nucleic acids and live organisms [2]. The goal of ideal drug delivery maximizes accumulation of loading therapeutics at the target site without off-target leakage during transporting [2]. Driven by advances in nanotechnology, either utilizing nanomaterials for drug delivery [3,4] or converting the drugs themselves into nanoscale therapeutic agents can enhance accumulation in the target region through their specific size [5]. However, the fixed physicochemical characteristics (such as shape, size, and charge) of traditional nanocarriers cannot adapt to the contradictory physicochemical requirements of multiple biological barriers. For instance, upon intravenous injection, nanoparticles (NPs) need to remain electrically neutral or negative during blood circulation, conversely, positively charged NPs will enter the cell more readily [6]. Essentially, it is difficult for nanocarriers with invariable physicochemical properties to overcome multiple biological barriers for efficient drug delivery. Thus, there is a pressing demand for developing smart nanomaterials with adaptive change of its physiochemical properties at different delivery stages.

Beyond the demand for advanced drug delivery systems, peptides have emerged as ideal building blocks for constructing smart nanomaterials due to their unique biological and structural properties. Peptides are a class of biomolecules possessing high biocompatibility [7,8], ease of synthesis and modification [9], showing great potential for biomedicine [10]. Directly as therapeutic agents, peptides are unique drugs that occupy a distinct position in the treatment of certain diseases, like insulin for diabetes [11]. Additionally, supramolecular peptide self-assembly, a bottom-up approach, can be employed to fabricate nanomaterials [12]. Self-assembling peptide nanomaterials have many attractive advantages, such as high biocompatibility, easy functionalization and structural programmability [13]. Peptide self-assembly is a spontaneous process in which the resultant nanostructures are determined and stabilized by a variety of intermolecular noncovalent interactions, including hydrophobic interactions, electrostatic interactions, hydrogen bonding, and van der Waals forces [14]. It is the nature of these weak interactions that these supramolecular nanostructures of peptide-based assemblies can be dynamically regulated and controlled with assembling molecular structures and the modulation of assembly environment, such as pH, temperature, and ionic strength. As a result, the structural programmability offers the groundwork for the creation of peptide-based supramolecular nanomaterials with stimuli-responsive morphological transformation. Notably, the dynamic adjustability of peptide assemblies enables them to respond to specific microenvironmental cues at pathological sites, which are distinct from healthy tissues.

Unlike healthy tissues, aberrant pH, aberrant redox gradient state, and overexpressed enzymes within pathological sites [15] can serve as biological signals to trigger the morphological transformation of nanocarriers. Stimuli-triggered morphologically transformable peptide nanomaterials integrate biological stimuli-responsiveness and morphological transformability of supramolecular assemblies. They not only enable targeted drug release, but also promote drug tissue penetration and retention via stimuli-induced morphological shifts, thereby optimizing therapeutic efficacy. In addition, there is another class of stimuli-triggered peptide nanomaterials directly as nanotherapeutics for treating diseases through morphological transformation strategy without the need for additional drugs.

**Fig. 1:**
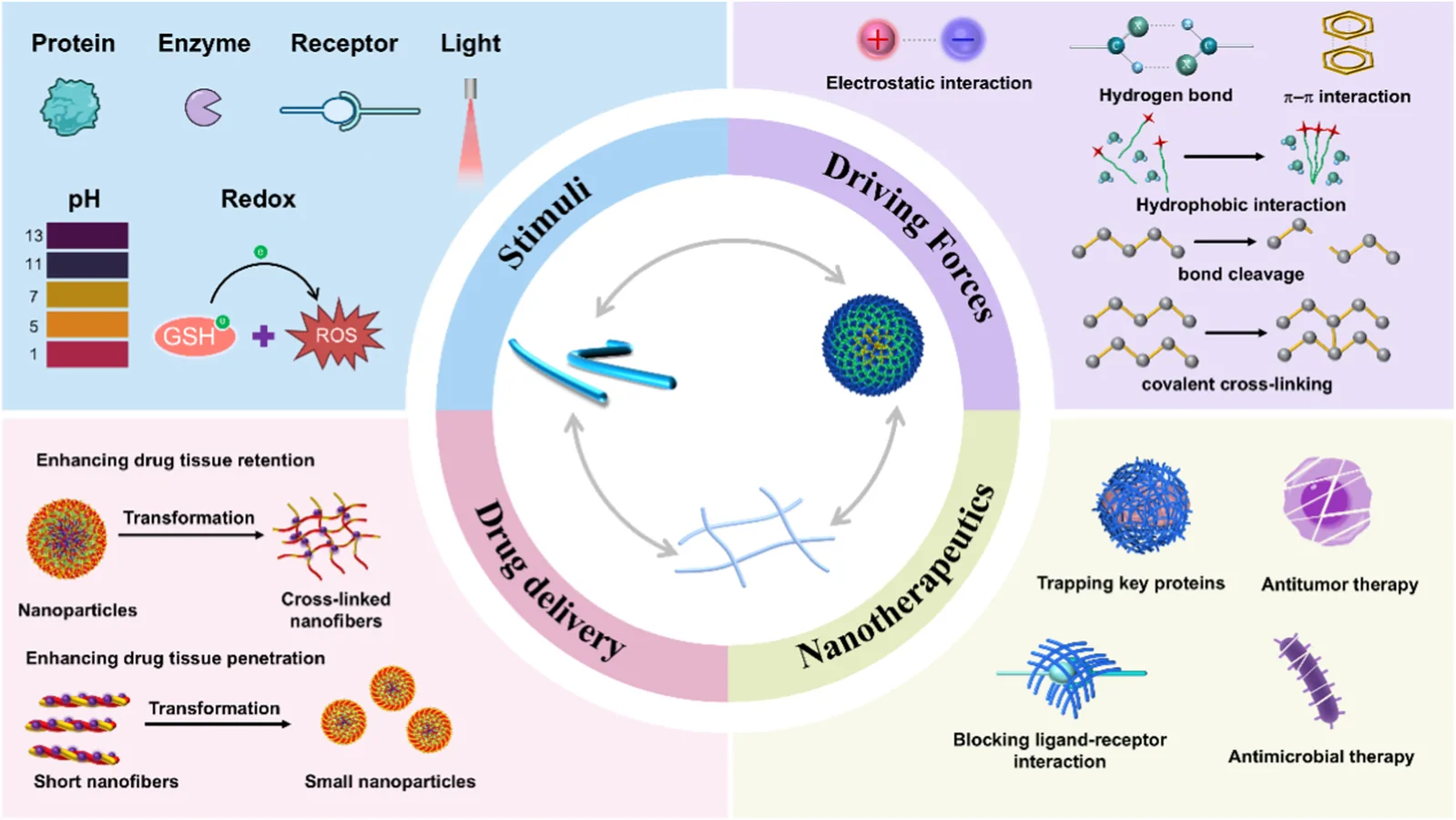
Illustration of morphologically transformable peptide nanomaterials, their stimuli, driving forces, and applications in drug delivery and nanotherapeutics.

In this review, we summarize recent progress of smart peptide nanomaterials with in vivo stimuli-responsive morphological transformation for biomedical application (Fig. 1). This review follows a logic of design principles, driving forces, in vivo stimuli, and applications, focusing on modular design strategies and the functional fitness between morphological transformation directions and biomedical needs. Two types of forces driving the morphological transformation of peptide nanomaterials are introduced, including noncovalent interactions and covalent interactions. The in vivo stimuli for triggering morphological transformation of peptide nanomaterials are also described. The molecular design, stimulus responsiveness and specific morphological transformations of these nanomaterials for drug delivery and employed as nanotherapeutics are emphasized along with the unique advantages in disease treatment. Finally, we propose challenges and opportunities for the utilization of morphologically transformable peptide nanomaterials for disease treatment to advance design and development of next-generation smart drug delivery vehicles and nanotherapeutics.

## 2 Forces driving the morphological transformation of peptide nanomaterials

Nanostructures by self-assembly of peptide-based monomers are determined by supramolecular noncovalent interactions, which are mainly influenced by the physiochemical characters of assembling monomers and external environment [16]. Changes in the external environment significantly influence the composition of noncovalent and covalent interactions, providing a theoretical basis for designing the peptide nanomaterials with in vivo stimuli-responsive morphological transformation. In this section, we focus on two types of forces driving the morphological transformation of peptide nanomaterials.

### 2.1 Noncovalent interactions

Nanostructures of supramolecular peptide nanomaterials are formed and stabilized by the cooperativity of various noncovalent interactions, including hydrophobic, electrostatic, host-guest, hydrogen-bonding, π–stacking, and van der Waals interactions. Owing to the relatively weak nature of these interactions, peptide assemblies are relatively susceptible to many factors, such as pH, concentrations, and temperature [17]. Essentially, morphological transition is a process in which the balance between the original noncovalent interactions is broken and a new balance is rebuilt. Based on amino acids with diverse molecular characteristics, peptide-based assembly motifs offer an enriched array of noncovalent interaction sites [18]. The amide bond (-CO-NH-) on the peptide backbone serves as an ideal site for forming oriented hydrogen bonds, which are crucial for the formation and stabilization of the secondary structure of the peptide chain [19]. Electrostatic interactions primarily arise from charged amino acid side chains, such as negatively charged aspartic acid and positively charged arginine. They can generate both attraction (opposite charges) and repulsion (same charges), playing a key role in regulating the stability, structure, and responsiveness of the assembly. Hydrophobic interactions refer to the rapid formation of disordered aggregates by hydrophobic motifs to avoid water [20]. They are the key driving force for the assembly of peptide monomers into micelles or vesicles. π-π stacking mainly occurs between molecules containing aromatic amino acids [21], such as phenylalanine and tyrosine, and is crucial for stabilizing the fiber morphology and forming an ordered hierarchical morphology [22]. Non-natural aromatic conjugated groups can also serve as important sources of π-π interactions. Tuning π–π interactions through the incorporation of aromatic groups, while preserving the peptide sequence, enables control over assembly morphology [23]. In recent studies, the incorporation of synthetic aromatic groups has significantly enhanced π-π stacking, altering the driving forces and pathways of peptide assembly as well as the nanoscale morphology, thereby yielding unique supramolecular structures and materials [24]. Van der Waals forces are weak, ubiquitous forces that often cooperate with other noncovalent forces to influence the self-assembly process.

Electrostatic interactions, hydrophobic effect, and hydrogen bonding emerge as particularly predominant and efficacious triggers for driving morphological transformations of peptide nanomaterials, especially within the complex biological systems. Their superiority lies in their pronounced sensitivity to the subtle yet critical changes in the physiological landscape, enabling precise, spatiotemporal control over nanomaterial morphology and function. Electrostatic interactions, arising from the attraction between oppositely charged amino acid side chains, are exquisitely tunable by the local ionic environment. The protonation state of these acidic and basic residues is highly dependent on local pH. The mildly acidic microenvironment characteristic of pathological sites, such as tumors [25] or inflammatory sites [26], can neutralize negatively charged carboxylate groups, effectively weakening electrostatic repulsive forces. This shift in the net molecular charge can destabilize an existing assembly or induce a conformational switch that favors an alternative, more stable nanostructure under the new charge distribution. The hydrophobic effect, arguably the dominant driver for the folding of globular proteins [27] and the assembly of amphiphilic peptides [28], is equally powerful in dictating the morphological fate of peptide nanomaterials in vivo. Mechanistically, in an aqueous environment, hydrophobic domains tend to sequester themselves from water, leading to the formation of micelles, vesicles, or fibrillar morphologies. This driving force is intensely sensitive to changes in solvation and polarity. In vivo key triggers include enzymatic cleavage and redox potential. Enzymatic cleavage involves the hydrolysis of a specific peptide bond by overexpressed enzymes, such as matrix metalloproteinases (MMPs) and cathepsins, which can remove a hydrophilic segment dramatically altering the overall amphiphilicity of peptides and triggering assembly or transformation. Redox-related triggers involve the reduction of disulfide bonds (-S-S-), which are often used to link hydrophobic moieties. Elevated intracellular glutathione (GSH) mediates this reduction, exposing hydrophobic domains and inducing aggregation or morphological transformation. Additionally, hydrogen bonds exhibit high selectivity and directionality. In vitro, by altering solvents, hydrogen bonds can serve as the dominant factor regulating peptide assembly processes and inducing morphological changes in the resulting assemblies [29]. In vivo, when triggered by biological recognition signals such as receptor-ligand interactions, hydrogen bonding may act as a key driving force for inducing morphological transformation.

### 2.2 Covalent interactions

While noncovalent interactions play a dominant role in mediating reversible morphological transformations, covalent interactions also contribute to stimuli-triggered structural reconfiguration through more stable chemical modifications. Covalent interactions refer to covalent bonds within molecules, which are significantly stronger and more stable than dynamic, reversible noncovalent interactions. Covalent bonds, characterized by their high bond energy, form the backbone of peptide molecules, defining their primary structure and inherent conformational preferences. Covalently triggered morphological transformation primarily involves two distinct yet complementary ways, chemical bond cleavage and covalent cross-linking. Chemical bond cleavage involves the incorporation of chemically labile bonds or the peptide sequence within the monomers. These bonds remain inert during the initial self-assembly process but can be selectively cleaved by specific external stimuli, leading to a fundamental change in the molecular structure of the building block. This change, in turn, disrupts the existing noncovalent balance and drives the formation of a new stable nanostructure. Mechanistically, the cleavage alters monomers molecular properties such as hydrophobicity, or polarity.

In contrast to bond cleavage, covalent cross-linking is a process that introduces new covalent bonds between pre-assembled building blocks. The mechanism by which covalent cross-linking drives morphological transformation resembles chemical bond cleavage. Cross-linking induces assembly monomers into new molecular entities, shifts the noncovalent equilibrium, and thus leads to morphological transformation. As an example, the oxidation of thiols to disulfides causes cysteine-containing peptides to form dimers, leading to a transition from NPs to nanofibers (NFs) [30]. Notably, covalent interactions are widely applied in in vivo-responsive designs, such as disulfide bond cleavage in tumor high-GSH environments and reactive oxygen species (ROS)-induced cross-linking, which have been integrated into drug delivery and nanotherapeutic systems.

## 3 Stimuli for triggering in vivo morphological transformation of peptide nanomaterials

The morphological transformation of peptide nanomaterials in vivo is precisely regulated by specific stimuli, including biological or external signals. These stimuli act as molecular switches that alter the noncovalent and/or covalent interactions driving peptide self-assembly, thereby inducing dynamic morphological rearrangements. Notably, most stimuli are endogenous biological signals derived from the pathological microenvironment of diseases, such as abnormal pH gradients, overexpressed enzymes, redox imbalance, dysregulated ligand-receptor interactions (LRIs), and pathology-related biomolecules (e.g., pathogenic proteins, spermine). In addition, exogenous stimuli like light are also employed for spatiotemporally controllable morphological regulation due to their non-invasive and precise targeting advantages. Collectively, these stimuli enable peptide nanomaterials to adapt to the complex in vivo microenvironment, overcoming biological barriers and optimizing therapeutic outcomes.

### 3.1 pH

The intrinsic pH gradient between pathological and physiological microenvironments serves as a key endogenous stimulus inducing morphological transformation in peptide nanomaterials. Normal physiological pH is approximately 7.4, whereas tumor tissues exhibit a weakly acidic extracellular environment (pH 6.5–7.2) due to the Warburg effect, with even stronger acidity within intracellular endosomes/lysosomes (pH 4.5–6.5) [26]. Moreover, biofilm microenvironment within infectious sites is acidic (pH 4.5–6.5) [31]. This pH gradient provides an ideal target for precise therapy of nanomaterials. Peptide nanomaterials primarily respond to this acidic stimulus through two molecular mechanisms. First, protonation-induced hydrophilic-hydrophobic balance transitions occur when ionizable groups (e.g., histidine imidazole rings) in peptide chains undergo protonation under acidic conditions. This disrupts intermolecular interaction equilibria, driving self-assembly into nanostructures. For instance, FA-EEYSV-NH_2_, a folic acid-conjugated peptide with glutamic acid residues, self-assembles into NPs at physiological pH (7.0) but reorganizes into NFs under acidic conditions (pH 5.0) [32]. Second, the cleavage of acid-sensitive chemical bonds, such as imine bonds, which hydrolyze under acidic conditions, leading to the transition of hydrophilicity of assembled monomers, ultimately resulting in morphological transformation. Accordingly, precisely manipulating the morphological transitions of peptide-based materials is an effective strategy by utilizing the specific pH of pathological sites within the body.

### 3.2 Enzyme

Similar to pH stimulation, specific enzymes overexpressed in vivo represent another class of key biological signals that induce morphological transitions in peptide nanomaterials. In numerous pathological sites, particularly within the tumor microenvironment, multiple enzymes, such as MMPs, and alkaline phosphatase (ALP), exhibit abnormally high expression [33]. This dysregulation provides highly specific, efficient endogenous triggers targets for designing intelligent drug delivery systems. The core of enzyme-responsive peptide-based material design lies in incorporating enzyme-recognizable specific substrates, such as specific peptide sequences or chemical bonds. The morphological transformation of enzyme-triggered peptide nanomaterials is induced by altering the hydrophilicity, hydrophobicity, or charge of the materials through enzymatic reactions.

Among various enzyme-responsive systems, the ALP-responsive system has been extensively explored. ALP is upregulated in various cancers [34], this enzyme can dephosphorylate a variety of phosphorylated substrate, including phosphorylated proteins and nucleotides [35]. In the development of ALP-responsive peptide nanomaterials, phosphorylated tyrosine is of particular interest, not only because it is a substrate for ALP, but also because the aromatic tyrosine side chain provides additional hydrophobicity and π–π stacking interactions after dephosphorylation. The introduction of a phosphate group increases the hydrophilicity and electrostatic repulsion of the peptide monomers, thereby helping to maintain a good state of dispersion or metastability. During the ALP-catalyzed dephosphorylation process, phosphorylated tyrosine residues are converted into hydrophobic tyrosine residues, which disrupts the amphiphilic equilibrium and thereby promotes self-assembly or morphological transformation. Therefore, the incorporation of phosphorylated tyrosine is a simple and commonly used strategy for constructing ALP-responsive peptide-based nanomaterials for cancer therapy. For instance, doxorubicin (Dox)-loaded NPs composed of the amphiphilic peptide IpYR incorporating phosphotyrosine were cleaved by overexpressed ALP at tumor sites, thereby transforming into NFs that effectively prolong drug retention at tumor sites in vivo more than the control peptide without morphological transformation ability [36].

### 3.3 Redox

Redox potential gradients exhibit significant variations at cellular and tissue levels [37], making them a crucial endogenous stimulus for inducing morphological transformations and functional activation of nanomaterials in vivo. This phenomenon is particularly pronounced in the tumor microenvironment, characterized by high concentrations of the reducing agent GSH and active ROS [38]. Regarding reductive responses, GSH, a key intracellular reducing agent, exhibits cytoplasmic concentrations significantly higher than those in the extracellular fluid, up to 100–1000 folds [39]. Moreover, GSH levels within tumor tissues are markedly elevated compared to normal tissues. The most classic design utilizes disulfide bonds as the responsive unit [40]. Disulfide bonds remain stable in normal extracellular environments but rapidly reduce and cleave into thiol groups upon endocytosis and exposure to high GSH disease environments. This process strips the hydrophilic shell from nanomaterials, triggering morphological transformation.

Overexpressed ROS constitute another critical redox stimulus. ROS primarily include hydrogen peroxide (H_2_O_2_), superoxide anion (O_2_^−^), and hydroxyl radical (·OH) [41]. In pathological states such as tumors or inflamed tissues, ROS levels significantly exceed those in healthy tissues [42]. This oxidative stress not only promotes tumor progression and drug resistance [43], but also provides the basis for designing ROS-triggered morphological transformation materials. The mechanisms triggering morphological transformation fall into two primary categories. First, ROS-induced chemical bond cleavage, where bonds like thioether and borate bonds undergo irreversible breakage under ROS exposure, inducing hydrophilic-hydrophobic imbalance that drive morphological transitions. Second, ROS-induced covalent cross-linking. For instance, a cysteine-containing peptide amphiphile transformed into disulfide-crosslinked dimers in oxidative microenvironments, causing their transition from NPs to NFs [30]. These ROS-responsive materials not only enable targeted delivery but also possess intrinsic ROS-consuming properties that help regulate imbalanced oxidative stress microenvironments, delivering additional therapeutic benefits.

### 3.4 LRIs

Intercellular communication through LRIs serves as a fundamental mechanism [44] governing biological processes in multicellular organisms, including organogenesis, tissue repair, and immune regulation [45]. Dysregulation of these molecular recognition events frequently correlates with pathological progression, rendering targeted LRI modulation a promising therapeutic strategy.

Beyond chemical signals, LRIs based on biorecognition represent another class of highly specific stimuli that induce precise morphological transformations of peptide nanomaterials in vivo. For instance, tumor cell surfaces often overexpress specific receptors such as integrins, human epidermal growth factor receptor 2 (HER2), endothelial growth factor receptor (EGFR), and programmed cell death protein 1 (PD-1) [[46], [47], [48]]. In rheumatoid arthritis synovium, pro-inflammatory macrophages overexpress CD44 and folate receptors on their surfaces [49]. By embedding matching target ligands within the designed peptide sequence, peptide materials can actively recognize these receptors and accumulate on the target cell surfaces. This high-affinity binding interaction triggers in situ morphological transformation of the peptide nanomaterials from NPs to NFs. The fibrous network formed by these NFs physically blocks receptor-mediated biological functions, thereby exerting therapeutic effects. This strategy of biorecognition-triggered morphological transformation enables nanotherapeutics that can show therapeutic action without relying on drugs.

### 3.5 Light

Light, as an exogenous stimulus characterized by its non-invasive nature and exceptional spatiotemporal resolution [50], presents a powerful tool for precisely controlling the morphological transformations of peptide nanomaterials. The main strategy involves the strategic incorporation of photo-responsive moieties into the molecular design. Upon irradiation at specific wavelengths, these moieties undergo photochemical reactions that directly alter the molecular structure of the peptide building blocks. A highly effective strategy utilizes a photo-triggered cascade reaction whereby irradiation activates the photosensitizer, prompting the generation of ROS, which in turn act as a secondary stimulus to oxidize labile motifs within the construct. This cascade acts as a switch, initiating a precisely tailored morphological transition at the nanoscale.

### 3.6 Other stimuli

Besides the aforementioned stimuli, other pathology-related biomolecules can also act as specific triggers for morphological transformation, expanding the scope of stimuli-responsive design. Pathology-related proteins (PRP) not only exhibit abnormal expression in pathological lesions but also directly participate in or regulate the pathophysiological processes of disease [[51], [52], [53]]. Thus, PRPs are key targets and used as stimuli. Abnormally expressed pathogenic proteins serve as crucial biological signals that trigger morphological transformations of peptide nanomaterials. Upon binding to pathogenic proteins, peptide nanomaterials containing targeted peptide sequences transition from NPs to NFs, encapsulating and trapping the proteins to block their mediated biological functions.

Spermine, a crucial endogenous polyamine overexpressed in numerous cancers [54], serves as a potential disease biomarker [55]. Furthermore, it plays a pivotal role in sustaining and reinforcing the tumor immunosuppressive microenvironment [56,57], thereby promoting resistance to antitumor therapies. From a supramolecular chemistry perspective, spermine can function as a guest molecule capable of reversible binding with the host molecule cucurbit [7]uril (CB [7]) [58]. This dynamic and reversible host–guest interaction provides a foundation for designing spermine-responsive, morphologically transformable peptide nanomaterials. In principle, specific residues within self-assembling peptides, such as phenylalanine, can form stable host–guest complexes with CB [7] [59]. The steric hindrance introduced by CB [7] binding disrupts the initial supramolecular assembly of the peptides, leading to a distinct assembled state. Subsequent competitive binding of spermine dissociates the CB [7]–peptide complex, releasing the peptides and triggering a morphological transformation.

## 4 Biomedical applications of in vivo stimuli-responsive morphologically transformable peptide nanomaterials

Morphologically transformable peptide nanomaterials have demonstrated versatile and promising biomedical applications, addressing key challenges in targeted therapy and disease intervention. These applications are primarily categorized into three core directions: drug delivery, standalone nanotherapeutics, and biomimetic nanotherapeutics, each capitalizing on stimulus-triggered morphological shifts to optimize therapeutic outcomes. In drug delivery, these nanomaterials act as smart carriers that overcome multiple biological barriers by transitioning between morphologies at specific delivery stages. NP-to-NF transformation mitigates elevated tumor interstitial pressure and vascular/hemorheological limitations to boost retention within tumor lesions. By contrast, NF-to-NP transformation overcomes the steric hindrance imposed by the dense extracellular matrix (ECM), facilitating deep tumor penetration and subsequent cellular internalization. As nanotherapeutics, they exert therapeutic effects directly through morphological transformation, without relying on external payloads, by blocking pathogenic protein/receptor interactions, disrupting cell membranes, or modulating intracellular signaling pathways. Additionally, biomimetic designs further enhance their performance by mimicking endogenous structures or biological processes, improving biocompatibility, targeting specificity, and in vivo retention.

### 4.1 Drug delivery

Drug delivery is the core application of in vivo stimuli-responsive morphologically transformable peptide nanomaterials. These smart nanocarriers are designed to adapt their morphology at specific stages of the delivery process by responding to endogenous or exogenous stimuli. The following sections categorize these delivery systems based on stimulus type and application scope, including single stimulus-triggered systems for cancer therapy, multiple stimuli-triggered systems for cancer therapy, and delivery systems tailored for other diseases beyond cancer. Across these categories, the core advantage lies in the ability of morphological transformation to optimize drug accumulation, prolong tissue retention, and improve therapeutic efficacy while minimizing adverse effects.

#### 4.1.1 Single stimulus-triggered drug delivery for cancer

Single stimulus-triggered drug delivery systems utilize one of the unique microenvironmental characteristics of tumor tissues or exogenous non-invasive signals as the trigger. By integrating corresponding responsive motifs into peptide-based nanocarriers, these systems achieve precise morphological transformation at tumor sites, thereby addressing key challenges in drug delivery such as targeted accumulation, long-term retention, and deep tissue penetration.

##### 4.1.1.1 pH-triggered systems

Local implantation of chemotherapy drugs enables long-term retention in tumor tissue while minimizing administration frequency and toxicity when compared to systemic treatment [60]. However, medical implants often resort to surgery, causing invasive harm to patients and increasing the risk of infection. An alternative solution is that smart morphologically transformable peptide-based vehicles enable long-term retention at tumor sites without invasive harm. A peptide-polymer conjugate BP-FFVLK(PEG)-H_6_ was designed, including a polyethylene glycol (PEG) chain for hydrophilicity, a hydrogen-bonding peptide Lys-Leu-Val-Phe-Phe (KLVFF) for β-sheet-like fiber formation, a hexapeptide composed of repetitive histidine for pH-responsiveness, and a bis-pyrene (BP) motif for π-conjugation and strong hydrophobicity [60]. The peptide-polymer conjugate self-assembled into NPs in phosphate-buffered saline (PBS) solution (pH = 7.4). Through the enhanced permeability and retention (EPR) effect, BP-FFVLK(PEG)-H_6_ NPs accumulated in tumor tissues following intravenous injection. Subsequently, histidine is protonated by the acidic environment, increasing the hydrophilicity of monomers. This promoted the breakdown of NPs and the transformation of NFs, which further interlinked to create a nested fiber network. The formed network allowed long retention of hydrophobic small molecules at the tumor site for up to 96 h via hydrophobic interaction. At 96 h after intravenous injection, the cumulative amount of indocyanine green (ICG) and Dox in tumor tissues of mice previously administered BP-FFVLK (PEG)-H_6_ NPs was more than twice that of mice without administration. This non-invasive implant not only enabled long-term retention of free drugs, but also had a high safety.

The peptide nanomaterials can be endowed with in vivo pH-triggered morphological transformation through acid-responsive bonds, apart from amino acid protonation. A such peptide-drug conjugate (DPC) was designed by conjugating Dox with a peptide KIGLFRWR [61]. When under neutral or slightly acidic conditions, morphology of the DPC assemblies underwent a time-dependent transition process from NPs to NFs. Importantly, the transformation process was controlled by electrostatic force. For intravenous injection, acid-responsive 2,3-dimethylmaleic anhydride (DMA) grafted polylysine (AR-PL) was used to interact with DPC, forming negative DPC-PL NPs. Based on acid-triggered morphological transition characterization, the DPC-PL NPs allowed for simultaneous targeting and long-term retention of tumor tissue. Compared to free Dox, DPC-PL NPs significantly increased concentration of the drug concentration in tumor tissue, while concurrently reducing its concentration in other tissues, particularly the heart and kidney. Additionally, mice treated with DPC-PL NPs had smaller tumor sizes and a higher survival rate compared to those treated with morphology-persistent liposomes loaded Dox or with Dox solution. Through further optimizing the peptide, a bifunctional peptide-drug conjugate D-ss-P was designed for dual inhibition of tumor growth and metastasis [62]. Dox was linked to the peptide CCKIGLFRWR capable of selectively inhibiting matrix metalloproteinase 2 (MMP-2) activity via disulfide bond, forming the conjugate D-ss-P. Similarly, positive D-ss-P and AR-PL formed stable D-ss-P@AR-PL NPs under physiological conditions via electrostatic force. After intravenous administration, D-ss-P@AR-PL NPs transformed into spherical network scaffolds on tumor cell membrane, enhancing the retention of Dox at tumor sites.

In addition to morphological transition from NPs to NFs, the opposite shift from NFs to NPs can also be accomplished. The pentapeptide FF-Amp-FF (AmpF) containing the non-canonical amino acid 4-amino proline (Amp), transformed from NFs (pH = 7.4) to NPs (pH = 6.5) when the pH changed [63]. The reversible morphological change of AmpF is primarily attributed to the pH-dependent isomerization of the amide bonds in Amp. Two pH-responsive peptide-drug conjugates, AmpF-ss-CPT (disulfide-linked camptothecin) and AmpF-IR820 (photosensitizer IR820-conjugated) were developed, which co-assemble with AmpF to form the nanocarrier (ACI) with morphological transformation [63]. ACI transformed from NPs in acidic endo/lysosomes to NFs in neutral cytoplasm, as evidenced by bio-TEM. Pharmacokinetic studies revealed superior elimination half-life of ACI, attributed to its high-aspect-ratio NFs morphology that enhanced circulatory persistence and tumor accumulation. The dynamic morphological transformation of ACI significantly potentiated its therapeutic efficacy in combined chemo-photodynamic therapy. Likewise, Li et al. engineered pH-responsive peptide-drug conjugates, AmpF-ss-NLG919 (IDO1 inhibitor-loaded) and tyrosine-functionalized AmpFY, which co-assembled with AmpF into a morphologically transformable nanocarrier (AmpFY9) [64]. Imaging of isotope ^125^I-labeled AmpFY9 revealed enhanced tumor accumulation. The dynamic morphological switching of AmpFY9 promoted prolonged tumor retention, synergistically enhancing immunosuppressive tumor microenvironment reversal and tumor growth inhibition.

Simultaneous delivery of multiple drugs can be facilely incorporated in the pH-triggered nanomaterials. For instance, pH-triggered morphologically transformable nanocarriers enabled combinatorial delivery of metformin and sunitinib to potentiate photodynamic therapy (PDT)-mediated immune activation [65]. The peptide amphiphile Ce6-pep-MET featured acid-labile conjugation of metformin (tumor immunosuppression modulator) to lysine residues of fiber-forming peptide FFAANPEEEK, with chlorin e6 (Ce6) as hydrophobic domains. Sunitinib encapsulation yielded SUN-NPA NPs that underwent tumor-specific acidic transformation where pH-responsive metformin liberation triggered NPs-to-NFs transition enabling sustained sunitinib release. This morphology-switchable system demonstrated spatiotemporal control of immunomodulators delivery, synergistically amplifying PDT efficacy through dual immunosuppression blockade and tumor microenvironment reprogramming.

##### 4.1.1.2 Enzyme-triggered systems

Enzyme-triggered morphologically transformable nanomaterials exploit dysregulated enzymes as key therapeutic targets by integrating cleavable peptide sequences into their design. For example, CPT-FLPR, comprising camptothecin (CPT), β-sheet-forming LVFF, cathepsin B-sensitive GFLG linker, PEG spacer, and RGD targeting motif, was engineered to enable tumor-specific activation [66]. Upon lysosomal cathepsin B (CtsB) cleavage, CPT-FLPR NPs transformed to sustained-release NFs, while GGGG-substituted CPT-GGPR (morphology-persistent control) retained NPs shape. In vivo studies demonstrated superior efficacy of CPT-FLPR in suppressing postoperative tumor recurrence compared to CPT-GGPR or free CPT. Additionally, in the quantitative analysis of tumor tissue, the CPT-FLPR group showed the highest CPT accumulation in the tumor, which was approximately 3.4 times and 1.7 times that of the free CPT group and the CPT-GGPR group, respectively, highlighting the critical role of enzyme-triggered morphological transformation in spatiotemporal drug delivery.

**Fig. 2:**
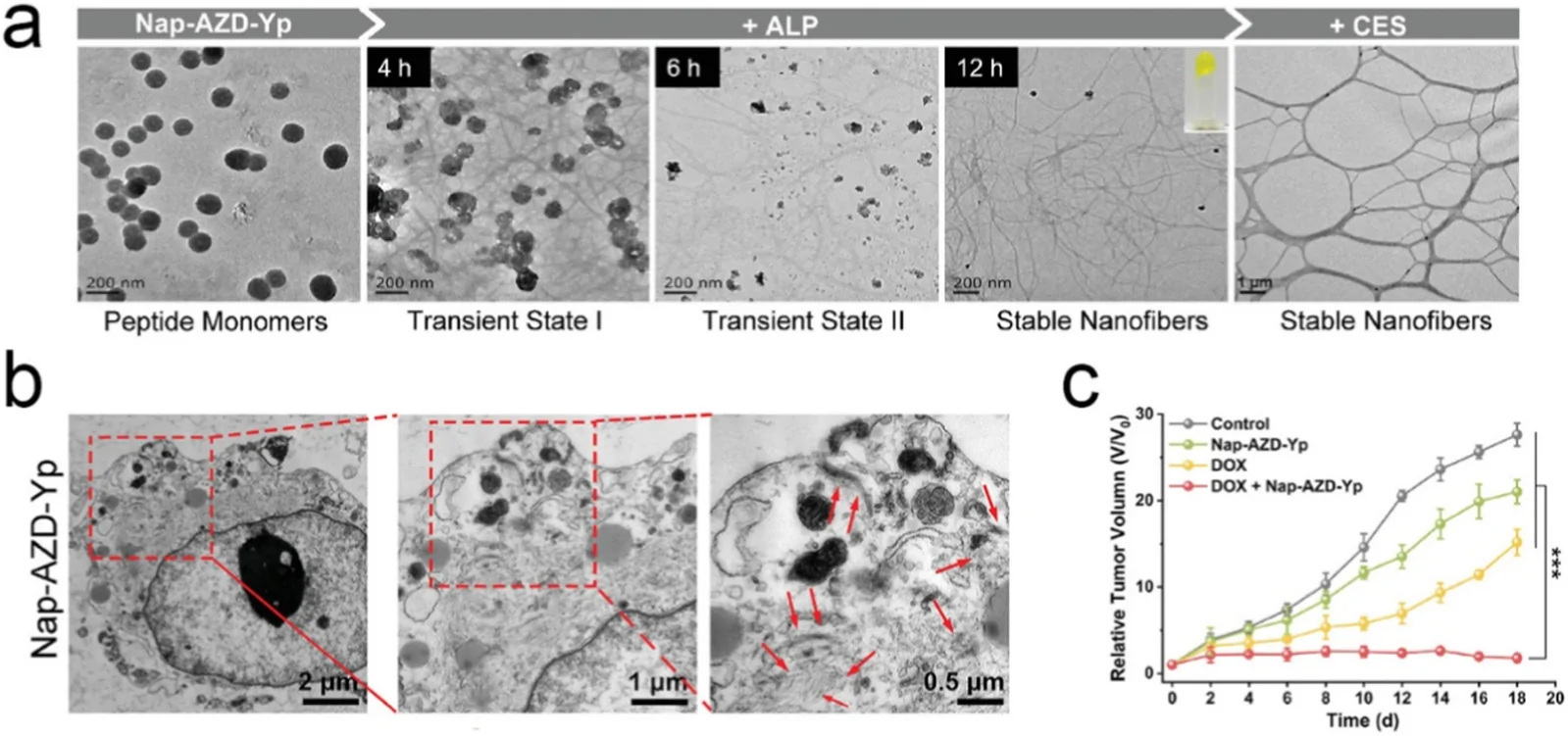
a) Enzyme-triggered transformations of Nap-AZD-Yp. b) TEM images of cancer cells treated with Nap-AZD-Yp. Red arrows indicate the NFs. c) Growth curves of tumors after different treatments. Reproduced with permission: Copyright 2022, Wiley-VCH GmbH [67]. (For interpretation of the references to colour in this figure legend, the reader is referred to the Web version of this article.)

Leveraging cancer-specific overexpression of ALP and carboxylesterase, a dual-enzyme-responsive nanocarrier Nap-FF-K(AZD8055)pY (Nap-AZD-Yp) was engineered for the delivery of autophagy-inducing AZD8055 [67]. This conjugate integrated self-assembling Nap-FF backbone, ALP-cleavable phosphorylated tyrosine, and carboxylesterase-labile ester linkage of AZD8055. Following cellular internalization, ALP-triggered dephosphorylation drove NPs-to-NFs transformation (Fig. 2a and b), prolonging intra-tumoral retention while carboxylesterase mediates sustained AZD8055 release. This morphology-switchable system synergized with Dox chemotherapy in vivo, demonstrating enhanced tumor suppression versus monotherapies (Fig. 2c) through autophagy-mediated chemo-sensitization and spatiotemporal control of drug release.

**Fig. 3:**
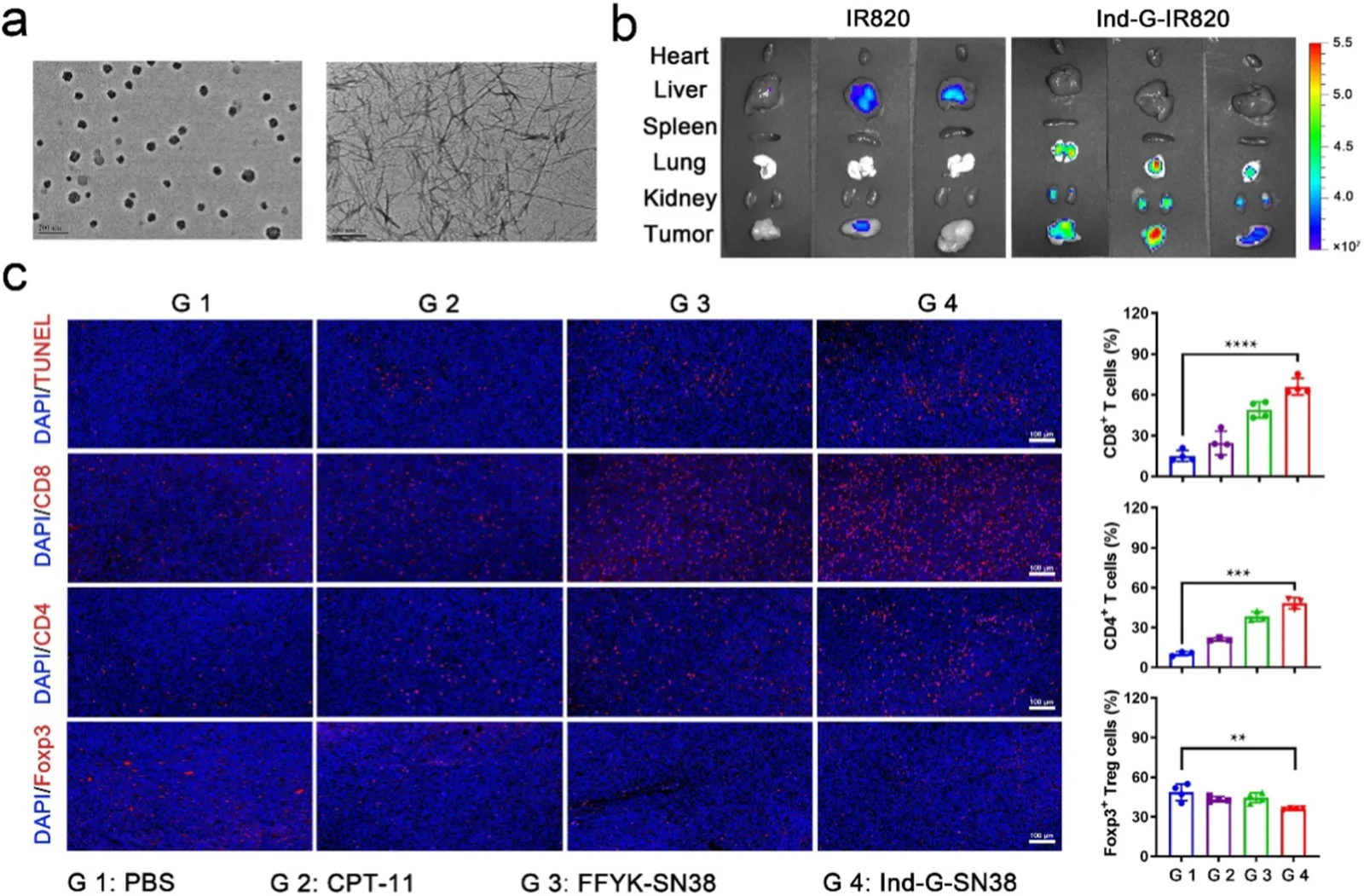
a) TEM images of the Ind-G-SN38 (left) and the Ind-G-SN38 treated by carboxylesterase (right). b) Ex vivo fluorescence images of the mice treated by IR820 and IR820-labed Ind-G-SN38. c) Immune cell infiltration and apoptosis in tumor tissues across different treatment groups. Reproduced from Ref. [68], licensed under CC BY 4.0.

Further optimization enabled enzyme-responsive nanocarriers to achieve co-delivery of multiple drugs. A peptide–drug conjugate, Ind-G-SN38, was constructed to deliver the chemotherapeutic agent SN38 (7-ethyl-10-hydroxycamptothecin) together with indoximod, an inhibitor of indoleamine 2,3-dioxygenase, for enhanced chemo-immunotherapy [68]. In this conjugate, SN38 was linked via an ester bond, while indoximod was conjugated through an amide bond. The self-assembled Ind-G-SN38 NPs can be hydrolyzed by carboxylesterase overexpressed in the tumor microenvironment, releasing SN38 and Ind-GFFYK which undergoing in situ transformation into NFs (Fig. 3a). These NFs improved local retention of indoximod (Fig. 3b), thereby amplifying its immunostimulatory effect (Fig. 3c).

**Fig. 4:**
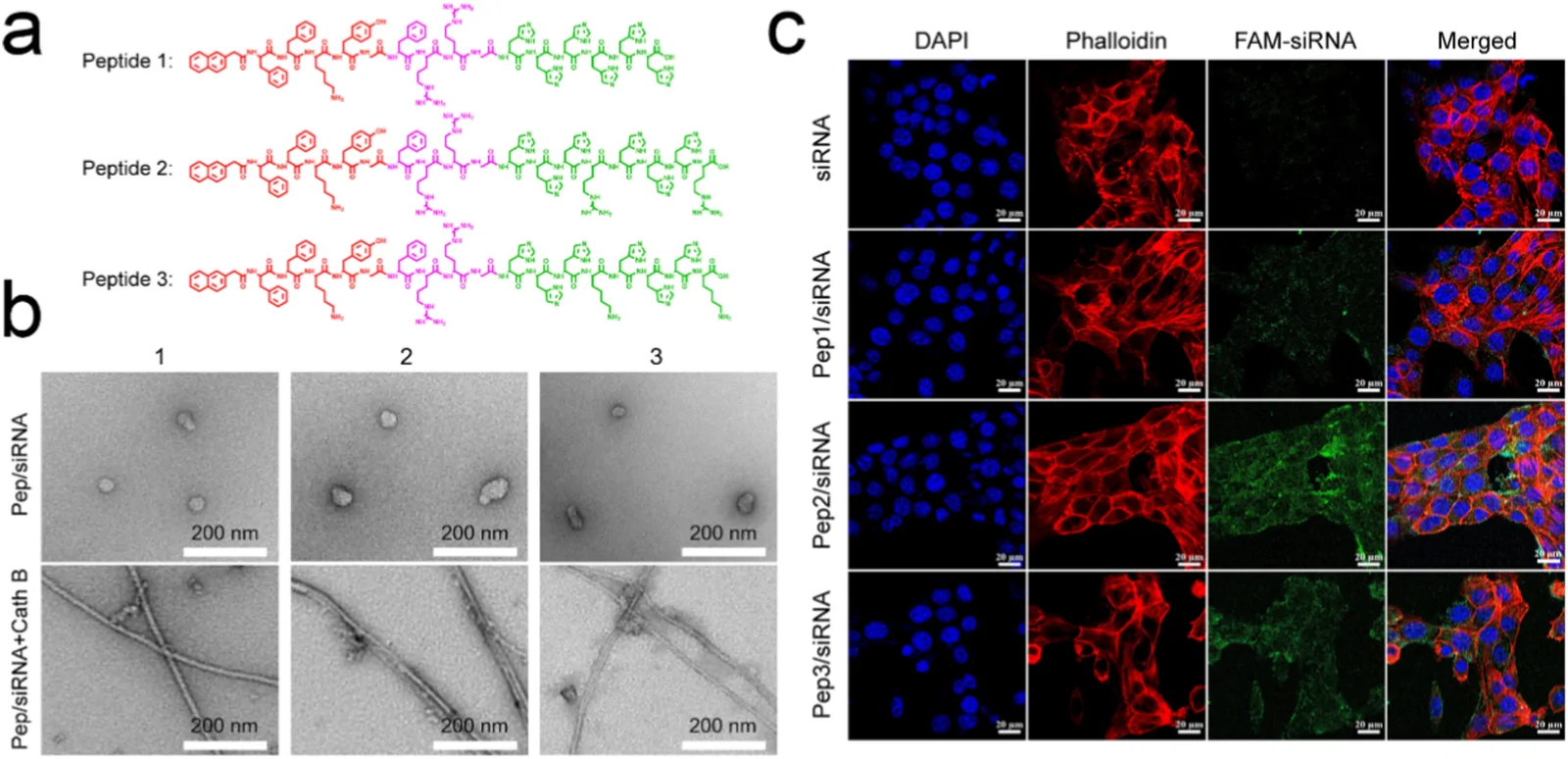
a) The chemical structures of three peptides. b) TEM images of untreated and CtsB-treated peptide/siRNA complexes. c) Intracellular delivery of siRNA by various peptide/siRNA complexes. Reproduced with permission: Copyright 2025, Elsevier [69].

As a versatile carrier platform, enzyme-responsive peptide nanomaterials were also employed for nucleic acid therapeutic delivery. To achieve efficient intracellular small interfering RNA (siRNA) delivery, the amphiphilic peptides were constructed containing three functional modules, including an assembly motif Nap-FFKY, a CtsB-responsive motif FRRG, and a cationic peptide sequence for siRNA binding (Fig. 4a) [69]. The peptides and siRNA co-assembled into NPs (Fig. 4b) via electrostatic interactions. After endocytosis, the peptide/siRNA NPs within endosomes/lysosomes were hydrolyzed by highly expressed CtsB. The cleaved peptide products reassembled into NFs (Fig. 4b) due to enhanced hydrophobic interactions and thereby inducing membrane disruption and facilitating the release of residual peptide–siRNA complexes into the cytoplasm. The higher intracellular pH compared with that of endo-lysosomal compartments diminished the positive charge on peptide chains, weakens their interaction with siRNA, and thus facilitated intracellular siRNA release (Fig. 4c).

To further expand the function of the peptide nanomaterials, the strategy of co-assembly of multiple functional peptides was introduced into the carrier design. Peptide bearing a β-galactose moiety both recognizes the galectin-1 (Gal-1) receptor and is hydrolyzed by β-galactosidase. Considering overexpression of enzyme β-galactosidase and Gal-1 receptor in cancer cells, Tian et al. developed tumor-dual-targeted co-assembled nanocarriers through integration of β-galactose-functionalized peptides and therapeutic modules for spatiotemporally programmed drug release [70]. The bola-amphiphilic SgVE was engineered by β-galactose-serine substitution at the N-terminus of SVVVE (SVE), enabling simultaneous Gal-1 receptor targeting and β-galactosidase responsiveness. Co-assembly of SgVE with two functional derivatives, SIRI (SVE-disulfide-linked irinotecan (IRI)) and SPMI (SVE-MDM2-binding peptide for p53 activation), generated SgVEIP NFs. The initial short bundled NFs morphology transformed into elongated NFs upon lysosomal β-galactosidase cleavage. This enzyme-triggered morphological switch enhanced tumor retention while enabling dual therapeutic activation, including GSH-responsive IRI release inducing DNA damage, and liberated SPMI-mediated MDM2 inhibition to stabilize p53. The synergistic strategy demonstrated superior antitumor efficacy versus monotherapies, exemplifying programmable peptide co-assembly for multi-modal cancer intervention.

##### 4.1.1.3 Spermine-triggered systems

Macrocycle-based host–guest interactions can integrate multiple noncovalent interactions, such as hydrogen bonding and π–π stacking, to form stabilized host-guest complexes. Importantly, the reversible nature of macrocycle-based host–guest molecular recognition provides a foundation for achieving controlled biological function of proteins. For instance, 4-tert-butyl-L-phenylalanine (tBuF) was incorporated at functional interfaces of model proteins through gene editing techniques [71]. The specific recognition of tBuF by CB [7] induced proteins inhibition, but reversibly counteracted through competitive guest displacement. This principle extended to developing spermine-responsive nanocarriers via CB [7]-mediated supramolecular engineering. The spermine-triggered morphologically transformable nanocarrier was constructed through host-guest complexation between CB [7] and phenylalanine-containing peptide-CPT conjugates (FFVLK-CPT), forming stable NPs [59]. Spermine-triggered competitive binding disrupts CB [7]-CPT interactions, liberating free CPT conjugates that reorganize into microfibers. Stimuli-responsive morphological transitions were confirmed with selective intracellular microfiber formation observed in spermine-overexpressing tumor cells and corresponding in vivo morphological transformations detected in tumor sections. The supramolecular nanocarrier demonstrated enhanced tumor retention and superior antitumor efficacy compared to free CPT, validating the therapeutic advantage of stimuli-controlled assembly switching.

##### 4.1.1.4 Light-triggered systems

As an exogenous stimulus, light triggers morphological transformations indirectly. For example, a light-triggered nanocarrier was engineered through co-assembly of two amphiphilic peptides, Ce6-FFVLK-PEG (ROS-generating Ce6) and BR-FFVLK-PEG (bilirubin (BR) oxidized by ROS) [72]. These components co-assembled into spherical micelles encapsulating thioketal-linked paclitaxel dimer (PTX_2_-TK), endowing ROS-mediated hydrophobicity to hydrophilicity transition of BR. Irradiation-triggered morphological transformation to NFs occurred exclusively at 1:1 Ce6/BR ratio, indicating the necessity of optimal ROS-to-BR stoichiometry for morphological reconfiguration. In vivo studies demonstrated that light-activated NFs formation significantly improved tumor retention compared to non-irradiated spherical assemblies.

##### 4.1.1.5 LRI-triggered systems

Inspired by Bacillus Calmette-Guérin (BCG), which preferentially adheres to wounded urothelium through specific binding to exposed fibronectin [73], the binding-triggered, morphologically transformable nanocarrier C12-KF-NL was rationally engineered for enhanced bladder retention [74]. Comprising the fibronectin-binding peptide GNRQRWFVVWLG, the β-sheet-forming peptide KLVFF, and a hydrophobic dodecyl moiety, C12-KF-NL self-assembled into NPs with Dox encapsulated in their hydrophobic cores (Dox@C12-KF-NL). Following topical administration, these NPs bound exposed fibronectin in postoperative wounds, transforming into NFs that wrapped residual tumors for sustained Dox release. In vivo studies confirmed Dox@C12-KF-NL preserved an 8.5-fold higher Dox concentration in the bladders of mice compared to free Dox, 3 days following instillation.

#### 4.1.2 Multiple stimuli-triggered drug delivery for cancer

The complex in vivo microenvironment, characterized by multiple sequential biological barriers, poses significant challenges to single stimulus-triggered nanocarriers, which often struggle to achieve precise and efficient drug delivery due to insufficient spatiotemporal control. To address this limitation, multiple stimuli-triggered morphologically transformable peptide-based nanocarriers have been developed, leveraging the synergistic response to two or more endogenous or exogenous stimuli. This strategy enables stepwise and programmable morphological transformations at different stages of drug delivery, thereby overcoming sequential biological barriers, enhancing targeted accumulation, improving intracellular uptake, and achieving controlled drug release with high precision. Based on the response mode of stimuli, multiple stimuli-triggered systems are mainly classified into simultaneous triggers and sequential triggers, and some advanced designs even involve triple morphological transitions to further optimize therapeutic efficacy.

##### 4.1.2.1 Simultaneous triggers

Flufenamic acid (FA) was a small molecular drug activating of dysregulated Hippo pathway for inhibiting cancer cell proliferation and metastasis [75]. To construct dual-stimuli triggered morphologically transformable nanocarriers Fmoc-S(FA)FFYSV-SS-PEG_1000_(FASP), hydrophilic PEG chain was attached to the valine of self-assembling backbone Fmoc-SFFYSV via a disulfide bond, FA was linked to the serine with an Fmoc group via an ester bond [76]. After tumor cell internalization, the FASP NPs transformed into NFs, releasing FA which entered the nucleus and activated Hippo downstream pathway under dual action of overexpressed GSH and esterase. Moreover, the NFs containing bioactive peptide YSV increased the expression of MST1 kinase in the upstream of Hippo pathway, synergistically promoting Hippo pathway activation.

##### 4.1.2.2 Sequential triggers

The PEG-His@BPC nanocarriers were designed for the targeted delivery of berberrubine (BBR) via morphological transition from NPs to NFs upon sequential triggering by pH and MMP-2 [77]. The nanocarrier is composed of polyethylene glycol-histidine (PEG-His) and BBR-PLGVRKLVFF-Ce6 (BPC), where BPC integrates the hydrophilic anticancer drug BBR, photosensitizer Ce6, and the self-assembling MMP-2-responsive motif PLGVRKLVFF. BPC self-assembled into positively charged spherical NPs, which are not suitable for intravenous delivery. To address this limitation, the negatively charged PEG-His was coated onto the NPs via electrostatic interactions, resulting in the formation of the final PEG-His@BPC nanocarriers. Upon entering the acidic tumor microenvironment, PEG-His is detached through histidine protonation. The exposed BPC NPs then transform into NFs under the triggering of MMP-2, while simultaneously releasing BBR to induce tumor cell apoptosis. Additionally, the NFs accumulated at the tumor site generate a substantial amount of ROS, which kill cancer cells upon laser irradiation.

Intracellular and nuclear translocation of cargoes imposes distinct demands on the morphology of nanomaterials. Compared with morphologically invariant nanocarriers, multi-stimuli-responsive morphologically transformable nanocarriers exhibit superior nuclear-targeted delivery efficiency via spatiotemporally controlled morphological transitions. The peptide-drug conjugate HCPT-GFFpY-ss-ERGD (HpYss) was constructed with 10-hydroxycamptothecin (HCPT, an anticancer drug), self-assembling FF motifs, tumor-targeting RGD peptides, phosphorylated tyrosine (pY), and disulfide bonds [78]. Upon systemic administration, HpYss molecules accumulated at tumor tissues and were first triggered by extracellular ALP to self-assemble into NPs, which are readily endocytosed by tumor cells. Subsequently, the internalized NPs were further transformed into NFs under the stimulation of intracellular overexpressed GSH, facilitating nuclear delivery of HCPT. Benefiting from the precise nuclear targeting achieved through multi-stimuli-triggered morphological transformations, HpYss enables enhanced tumor accumulation (13.6-fold higher than free HCPT) and prolonged retention time of HCPT, thereby markedly enhancing its antitumor efficacy.

##### 4.1.2.3 Triple morphological transition

Multi-stimuli-responsive morphologically transformable nanomaterials can undergo triple morphological transitions in response to sequential stimuli. For instance, the MEL/Cypate@HA nanocarriers were composed of melittin (MEL, a non-selective cytotoxic peptide), the photothermal agent cypate, and hyaluronic acid (HA, a tumor-targeting polymer) [79]. Following systemic administration, MEL/Cypate@HA targeted tumor tissues and transformed from 50 nm NPs into a NFs network, which prolongs tumor retention. Notably, upon laser irradiation, the NFs were further converted into smaller NPs (25 nm), facilitating deep tumor penetration.

Multi-stimuli-triggered triple morphological transition nanocarriers can also deliver diverse small-molecule drugs. The peptide-drug conjugate Nap−CPT−HCQ−pY was constructed with three core components, the self-assembling backbone motif Nap-FFKK, pY linked to the C-terminus of the backbone peptide, CPT and autophagy inhibitor hydroxychloroquine (HCQ) conjugated to the lysine side chains via ester bonds [80]. In the absence of external stimuli, Nap−CPT−HCQ−pY self-assembled into nanobrushes. Following intratumoral injection, the nanobrushes first underwent sequential hydrolysis by ALP and carboxylesterase, transforming into NPs to enhance tumor cell endocytosis. Subsequently, the intracellular NPs further converted into Nap-Y NFs, with concurrent release of CPT and HCQ, which synergistically induced cancer cell death.

#### 4.1.3 Drug delivery for other diseases

Stimuli-responsive morphologically transformable peptide nanomaterials also show great potential in solving drug delivery problems for multiple other diseases. Pathological conditions including acute lung injury, bacterial infections, fundus neovascularization, and myocardial ischemia-reperfusion injury have unique microenvironmental characteristics. Abnormal enzyme expression, acidic biofilms and site-specific receptor overexpression can be used as triggers for precise morphological transformation. Structural adaptation to disease-specific stimuli allows these nanocarriers to overcome tissue-specific biological barriers, enhance local drug accumulation, prolong retention time, and reduce off-target toxicity.

##### 4.1.3.1 Acute lung injury

Enzyme-triggered morphological transformation of nanomaterials typically depends on enzyme overexpression, a key limitation to translational potential. To address this limitation, a self-amplifying feedback loop was engineered by coupling nanocarrier activation with endogenous enzyme upregulation. Capitalizing on the Nrf2/Keap1 pathway, in which nuclear factor erythroid 2-related factor 2 (Nrf2) governs NAD(P)H quinone dehydrogenase 1 (NQO1) expression, the NQO1-responsive peptide-based nanocarrier AmpFQB@Dex was developed [81]. This system comprises two quinone propionic acid (QPA)-modified peptides, AmpFQ (single QPA modification) and AmpFQ-ETGE (dual modification with QPA and the Keap1-binding ETGE peptide), which co-assemble with dexamethasone (Dex) into stable NPs. Upon inhalation, AmpFQB@Dex accumulated in inflamed lungs via the EPR effect, was internalized by inflammatory cells, and underwent NQO1-mediated transformation from NPs to NFs with Dex release. Liberated ETGE disrupted Nrf2-Keap1 complexes, activating Nrf2 to upregulate antioxidant enzymes (including NQO1) that scavenge Dex-induced ROS. Elevated NQO1 further amplified nanocarrier transformation, establishing a closed-loop cycle that enhanced local drug retention while mitigating ROS-mediated toxicity.

##### 4.1.3.2 Bacteria infection

To address Gram-negative bacterial outer membrane (OM) resistance, the morphologically transformable peptide-based nanocarrier NCefoTs was constructed by co-assembling lipopeptide ELp (inhibiting DsbA, a β-lactamase-stabilizing protein), lipopeptide MLp (targeting and disrupting OM), and cefoperazone (Cefo) [82]. ELp incorporated a palmitic acid moiety, β-sheet-forming FFVLA, a GGG spacer and a DsbA-inhibiting PFATCDF sequence, while MLp contained palmitic acid, a DsbA-cleavable disulfide bond, and lipopolysaccharide (LPS)-targeting membrane-disrupting KKRAKKFFKKPRVIGVSIPF peptide. Intravenous NCefoTs NPs recognized bacterial LPS, penetrated the OM, and selectively bound DsbA, triggering MLp cleavage via DsbA-mediated disulfide rupture. This released Cefo and induced NFs formation, enabling prolonged DsbA inhibition and enhanced antibiotic susceptibility of drug-resistant bacteria. In vivo sepsis models demonstrated NCefoTs significantly improve mouse survival rates compared to free Cefo, highlighting the efficacy of stimuli-responsive nanocarriers in overcoming microbial resistance barriers.

##### 4.1.3.3 Fundus neovascularization

To address ocular drug delivery challenges, a dual-functional system was engineered using the legumain-cleavable peptide AANLVFF (comprising β-sheet-forming LVFF and legumain-substrate AAN) [83]. Mannose and the cationic peptide motif RTT were employed to functionalize AANLVFF, yielding Mannose-AANLVFF (MP) for M2 macrophage targeting and RTT-AANLVFF (RP) for prolonged ocular surface retention, respectively. Co-assembly of MP, RP, and Dox generated MRP@Dox NPs that exhibited enhanced corneal retention through electrostatic adhesion and nanoscale barrier penetration. Upon uptake by M2 macrophages, legumain-mediated transformation of the NPs into NFs within lysosomes facilitated lysosomal escape and sustained release of Dox. This morphology transition synergistically induced apoptosis of pro-angiogenic M2 macrophages while suppressing pathological neovascularization.

##### 4.1.3.4 Myocardial ischemia-reperfusion injury

**Fig. 5:**
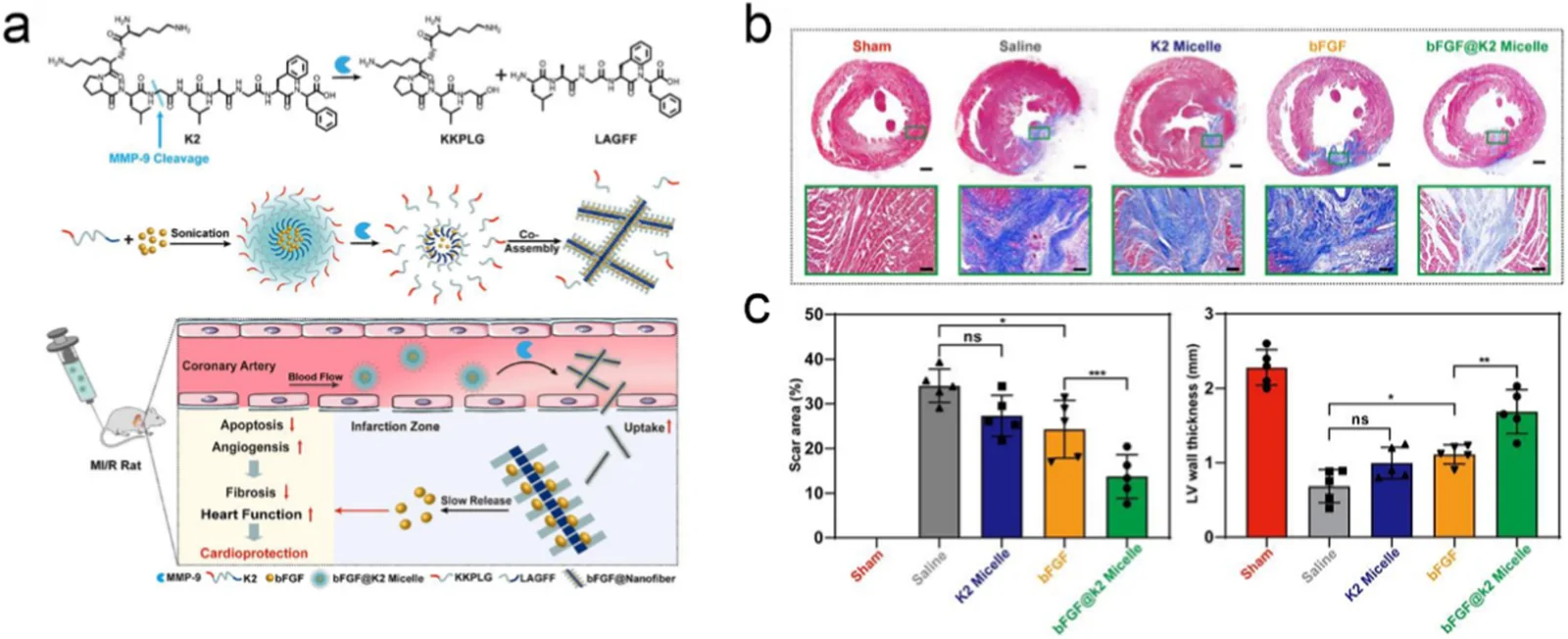
a) Schematic diagram of MMP-9-triggered morphologically transformable nanocarrier KPGF@bFGF for slowly release of bFGF at ischemic myocardium of myocardial ischemia-reperfusion rats. b) KPGF@bFGF significantly reduce myocardial fibrosis. c) KPGF@bFGF effectively reduced myocardial scar area and increased left ventricular wall thickness. Reproduced from Ref. [84], licensed under CC BY 4.0.

Ischemic myocardium-targeted KPGF@bFGF exploited MMP-9 overexpression and EPR effects [84]. The KPGF peptide composed of MMP-9-cleavable PLGLAG, FF assembly domain and hydrophilic KK formed micelles encapsulating basic fibroblast growth factors (bFGFs), forming the KPGF@bFGF (Fig. 5a). MMP-9-triggered spherical micelles-to-NFs transition in ischemic myocardium enabled sustained bFGF release (Fig. 5a), significantly reducing fibrosis and restoring cardiac function in myocardial ischemia-reperfusion models versus free bFGF (Fig. 5b and c). The peptide nanomaterials exemplify enzyme-guided morphological reprogramming for precision delivery of macromolecular therapeutics.

##### 4.1.3.5 Rheumatoid arthritis

**Fig. 6:**
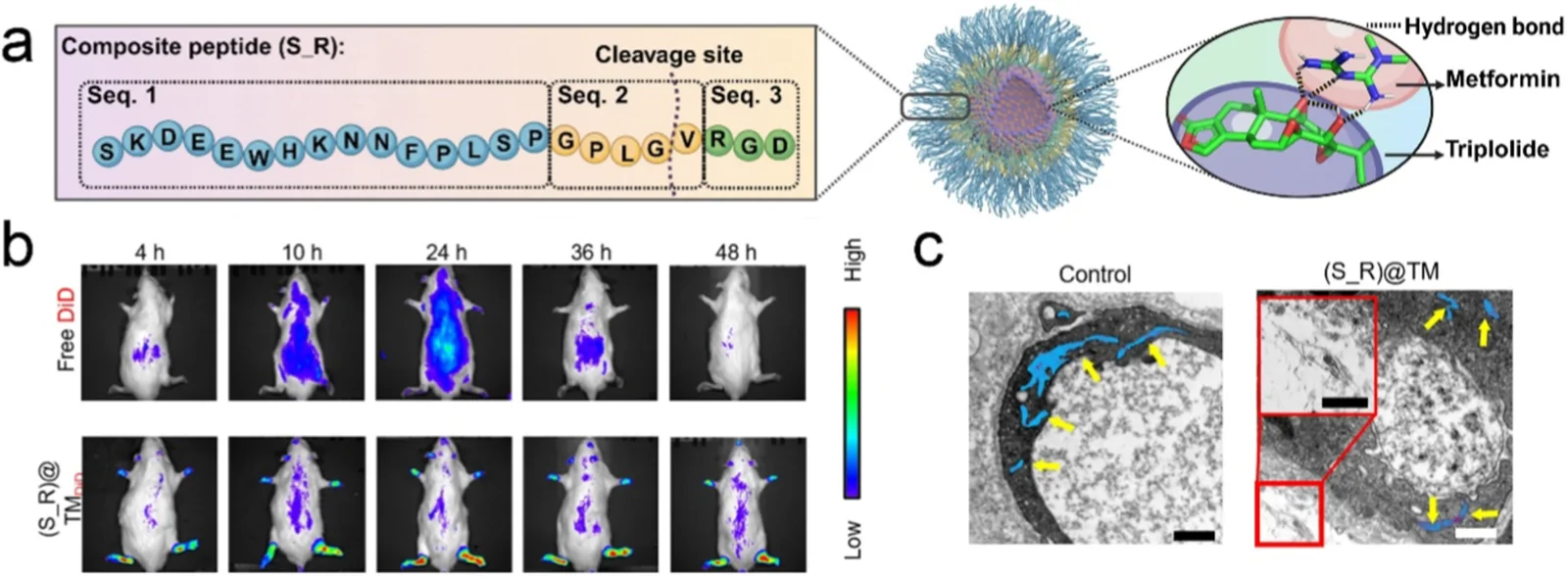
a) Schematic diagram of (S_R)@TM NPs. b) In vivo fluorescence images of arthritic rats treated with free dye and fluorescently labeled (S_R)@TM NPs. c) Bio-TEM images of rat synovium treated without or with (S_R)@TM NPs. red box indicates NFs. Reproduced with permission: Copyright 2025, AAAS [86]. (For interpretation of the references to colour in this figure legend, the reader is referred to the Web version of this article.)

Rheumatoid arthritis is a multifactorial autoimmune disease [85]. Monotherapy with single anti-inflammatory or immunomodulatory effects often shows limited efficacy, whereas multi-stimuli-responsive peptide nanomaterials enable comprehensive rheumatoid arthritis treatment via synergistic drug delivery and programmable morphological transformation. A functional peptide S_R, containing vascular endothelial growth factor receptor-2 (VEGFR2)-targeting, MMP2-cleavable and integrin αvβ3-binding motifs, was modified to prepare S_R-PEG_2000_-DSPE, which was co-assembled with triptolide and metformin into (S_R)@TM NPs (Fig. 6a) [86]. The NPs selectively accumulated in RA lesions by targeting VEGFR2 on inflamed joint endothelial cells (Fig. 6b). Cleaved by overexpressed MMP2, the NPs released peptide fragment STP-GPLG and formed cationic R@TM NPs with exposed RGD, which exhibited enhanced tissue penetration and were internalized by joint pathogenic cells. Notably, STP-GPLG self-assembled into an NFs network under acidic inflammatory conditions (Fig. 6c), which improved R@TM NP retention and inhibited inflammatory cell infiltration and pathological angiogenesis.

### 4.2 Nanotherapeutics

Peptide nanomaterials can be endowed with in vivo stimulus-triggered morphological transformation capabilities possess inherent therapeutic activities, eliminating the need for additional drug payloads. Unlike conventional nanocarriers that rely on loaded therapeutics to exert pharmacological effects, stimuli-responsive morphologically transformable peptide-based nanotherapeutics directly leverage dynamic morphological transitions to achieve therapeutic outcomes. Their therapeutic mechanism relies on the spatiotemporal control of nanostructure evolution in disease-specific microenvironments, thus enabling precise, targeted therapy with minimized off-target effects.

#### 4.2.1 Nanotherapeutics for cancer

Stimuli-responsive morphologically transformable peptide-based nanotherapeutics have emerged as a versatile platform for cancer therapy, leveraging the unique tumor microenvironment to trigger site-specific morphological changes. These nanotherapeutics are designed based on modular principles, integrating functional motifs such as stimuli-responsive segments, self-assembling cores, targeting ligands, and bioactive domains to achieve diverse therapeutic mechanisms. According to their action modes, they are mainly categorized into three types, including protein blocking type, receptor blocking type, and cytotoxic type.

##### 4.2.1.1 Protein blocking type of nanotherapeutics

For immunogenic ferroptosis induction in prostate cancer (PCa), TEP-FFG-CRApY integrated a photosensitizer (TPA-Eth-Py, TEP), peptide FFG, and the GSH peroxidase 4 (GPx4)-targeting peptide CRAWYQNpYCALRR (CRApY) [87]. The resulting nanomaterials showed ALP-dependent NPs-to-NFs transition. TEP-FFG-CRApY escaped from endosomes into the cytoplasm and increased intracellular ROS levels after laser irradiation. Mitochondrial crumpling was observed in cells treated with TEP-FFG-CRApY following irradiation. Mechanistically, ALP-responsive NFs formation induces lysosomal membrane permeabilization (LMP), releasing NFs that inactivate GPx4, a key factor in ferroptosis resistance, thereby enhancing iron-dependent cell death. In vivo studies demonstrated that TEP-FFG-CRApY effectively promoted dendritic cell (DC) maturation and cytotoxic T cell infiltration in PCa models.

The Warburg effect, a phenomenon whereby tumors predominantly convert glucose to lactic acid [88], has been shown to enhance both tumor proliferation and metastasis. Notably, renal cell carcinoma (RCC) exhibits a more pronounced intrinsic Warburg effect compared to other tumor types [89]. Based on this principle, PAC nanotherapeutics was designed to disrupt the Warburg effect by promoting pyruvate kinase M2 (PKM2) tetramerization [89]. Comprising β-N-acetylglucosamine-modified serine, KLVFF self-assembling peptides, and aggregation-induced emission fluorophores, PAC NPs accumulated in tumors. Here, O-GlcNAcase cleaved β-N-acetylglucosamine, converting NPs to NFs that exposed serine residues for PKM2 binding. This interaction inhibited glycolytic metabolism, improved antitumor efficacy in sunitinib-resistant models, and synergized with chemotherapy by reversing drug resistance.

Sulfatases are enzymes that catalyze the hydrolysis of sulfate ester-containing substrates [90] and are overexpressed in multiple tumor types [91]. Taking advantage of their pathological overexpression, sulfatase-triggered supramolecular proteolysis targeting chimeras (PROTACs) provide a novel strategy for cancer therapy [92]. The EIsYBE3 system combines the self-assembling capability of the bola-type amphiphilic hexapeptide EIYIYE (whose NFs formation is inhibited by sulfation) with PROTAC functionality to enable targeted protein degradation. EIsYBE3 was co-assembled from three components including sulfated tyrosine-containing EIsYIsYE (sY denotes sulfated tyrosine) and two EIYIYE derivatives (L1 and L2) modified with binding motifs. L1 bear the sequence GQVGRQLAIIGDDINR for selective recognition of the antiapoptotic protein Bcl-xL, while L2 incorporated LAHypYI to recruit the E3 ubiquitin ligase VHL. At optimized molar ratios, these components formed stable NPs in circulation. Upon intravenous administration, tumor-enriched sulfatases triggered desulfation of EIsYIsYE, inducing NPs-to-NFs transition. This morphological change exposed functional domains, promoting ternary complex formation between Bcl-xL, VHL, and EIsYBE3 NFs, thereby mediating ubiquitination and proteasomal degradation of Bcl-xL to induce tumor cell apoptosis. Characterized by precise enzyme-responsive activation and enhanced tumor retention, EIsYBE3 NPs exhibited superior target protein degradation efficiency. In vivo studies demonstrated potent inhibition of tumor growth, with synergistic effects when combined with the chemotherapeutic drug paclitaxel.

Microtubules (MTs) are key cytoskeletal components that play crucial roles in essential cellular processes such as cell division and intracellular transport [93]. Importantly, MT function is strongly dependent on dynamic properties [94,95]. Targeting tubulins, the core component of MTs, and disrupting their dynamic behavior constitute a clinically validated cancer therapy strategy [96]. Unlike conventional tubulin-binding agents that inhibit microtubule depolymerization, MT-targeting BP-FFVLK-RLPS NPs introduce a mechanism of inducing MT aggregation [97]. These NPs bond MTs via RLPS peptide-mediated tubulin recognition and underwent NFs formation to expose additional binding sites. These sites crosslinked MTs into stable hybrid aggregates. Further optimization involved conjugating the prostate-specific membrane antigen (PSMA)-targeting peptide YGWSFPYPQITG to lysine residues, yielding BP-FFVLK(YGWSFPYPQITG)-RLPS (BFYR) [98]. Intravenous BFYR NPs selectively targeted PCa through PSMA recognition, entered cells via endocytosis, and subsequently bound MTs. NFs formation induced apoptosis through cytoskeletal hyper-crosslinking. Moreover, BFYR suppressed androgen receptor transcriptional activation, synergistically inhibiting PCa progression.

Expanding beyond membrane receptors, BFV nanotherapeutics targeted intermediate filament dynamics through vimentin-binding VNTANST motifs [99]. Cellular internalization triggered vimentin-dependent NFs formation, disrupting skeletonization of vimentin and thereby reducing tumor cell migration. In vivo evaluation demonstrated significant suppression of pulmonary metastases in orthotopic breast cancer models.

##### 4.2.1.2 Receptor blocking type of nanotherapeutics

Targeting immune evasion, Cai and collaborators developed ALP-triggered PAC-SABI supramolecular assemblies to block CD47 and CD24 signaling [100]. The FFVLK(PEG_2000_)-AWSATWSNpYWRH monomers assembled into spherical micelles, subsequently the PEG segment was modified by CD24 antibody via michael addition reaction, yielding PAC-SABI NPs. Systemically administered PAC-SABI NPs targeted into tumor tissue where ALP hydrolysis converted NPs to NFs that crosslinked into a membrane-covering fiber network, simultaneously blocking CD47 and CD24. This enhanced macrophage phagocytosis and adaptive immunity, validated in breast and pancreatic cancer mouse models with potent antitumor responses. Gelatinases, including MMP-2/-9, are overexpressed at tumor tissue and play critical roles in cancer metastasis [101]. The MMP-2-cleavable peptide sequence PLGVRG [102] enabled the design of enzyme-responsive nanotherapeutics. The peptide-polymer conjugate PVA-CG-PD was engineered for tumor immunotherapy, comprising a PVA backbone, MMP-2-sensitive CGGKLVFFGPLGVRG-PEG, and a programmed cell death ligand 1 (PD-L1) antagonist peptide NYSKPTDRQYHF [103]. Following intravenous injection, tumor-targeted NPs shed their PEG shell via MMP-2 cleavage, disrupting amphiphilic balance to form NFs that exposed PD-L1 antagonists, potently inhibiting PD-1/PD-L1 immune checkpoint interactions.

The HER2 tyrosine kinase is a clinically relevant therapeutic target, as its overexpression in HER2^+^ malignancies drives tumorigenesis through constitutive dimerization-mediated signaling activation [45]. To address this, the LRI-triggered morphologically transformable nanotherapeutic TPM was engineered by incorporating three functional motifs, including AIE fluorophore BP, β-sheet-forming KLVFF sequence, and HER2-specific YCDGFYACYMDV peptides [104]. Upon HER2 binding, TPM underwent rapid NPs-to-NFs transition, with selective transformation observed on HER2-overexpressing cells. TPM significantly suppressed HER2 dimerization and subsequent downregulation of anti-apoptotic proteins. Notably, TPM exhibits prolonged tumor retention (>72 h) and achieves complete tumor regression in multiple HER2^+^ breast cancer xenograft models.

Anexelekto (AXL) is overexpressed on the cell membrane of ovarian cancer cells. Given its role in promoting cancer cell proliferation and invasion via diverse downstream signaling pathways upon dimerization [105], AXL-targeted Nap-IR NPs were engineered by conjugating the AXL-binding hexapeptide IRLRFK to self-assembling Nap-FF scaffolds [106]. AXL-triggered NPs-to-NFs transition on the ovarian cancer cell membrane was critical to the therapeutic efficacy. This morphological transformation shifted the apoptotic balance by downregulating the antiapoptotic protein BCL-2 and upregulating the pro-apoptotic protein BAX, achieving effective tumor growth inhibition as monotherapy. Combined with cisplatin, Nap-IR exhibited synergistic tumor suppression.

**Fig. 7:**
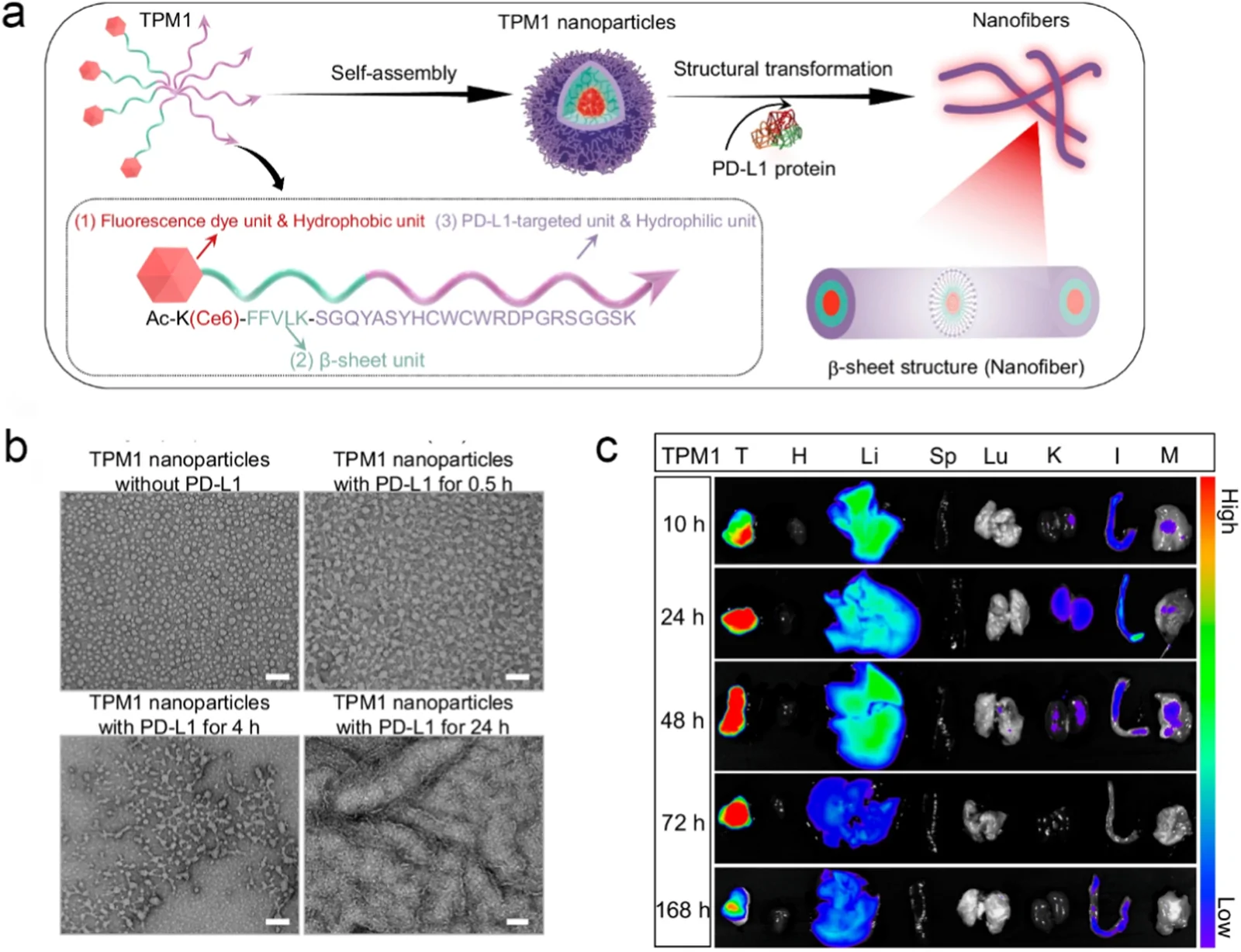
a) Schematic diagram of the molecular structure, fabrication process of TPM1, and its morphological changes triggered by PD-L1. b) TEM images of TPM1 NPs before and after incubation with PD-L1. c) Time-dependent in vivo fluorescence images of tumors and major organs after intravenous injection of TPM1 NPs. Reproduced with permission: Copyright 2024, Springer Nature [110].

Anti-PD-L1 monoclonal antibodies (mAbs) have been clinically applied for cancer immunotherapy by blocking the PD-1/PD-L1 pathway [107]. However, these mAbs are ineffective at targeting the majority of PD-L1 proteins, especially newly synthesized ones [108,109]. As an alternative approach for immune checkpoint inhibition, the TPM1 nanotherapeutic integrated a PD-L1-targeting peptide (SGQYASYHCWCWRDPGRSGGSK), a fluorescent Ce6 photosensitizer, and the self-assembling motif KLVFF (Fig. 7a) [110]. In vitro studies demonstrated that PD-L1 protein triggered the transition of NPs-to-NFs (Fig. 7b), whereas in vivo experiments revealed specific tumor accumulation of TPM1 NPs. These NPs formed fibrillar networks on cancer cell surfaces to block PD-1/PD-L1 interactions and reinvigorated CD8^+^ T cells. Notably, these NPs exhibited prolonged tumor retention (>7 days) (Fig. 7c), thus overcoming the key limitation of mAbs, which is their inability to effectively target newly synthesized PD-L1.

##### 4.2.1.3 Cytotoxic type of nanotherapeutics

PWG, a porphyrin-WG dipeptide conjugate, self-assembles into NPs at physiological pH (7.4) but reorganizes into NFs under acidic conditions (pH ≤ 6.5) [111]. Protonation of glycine carboxyl groups enhanced intermolecular hydrogen bonding, driving NPs-to-NFs transformation. Notably, NFs exhibited enhanced singlet oxygen (^1^O_2_) generation due to fibril-induced enhanced intersystem crossing, coupled with prolonged tumor retention (7 days in vivo), synergistically boosting PDT efficacy. Beyond oligopeptide sequences, chemical modifications have also been introduced to enable pH-triggered morphological reconfiguration. PEAK comprising protoporphyrin IX (PpIX) and DMA-modified (AEAEAKAK)_2_ formed NPs via DMA-mediated blockage of electrostatic interaction. Acidic tumor microenvironments cleaved DMA, exposing lysine charges to induce NPs-to-nanorods transitions [112]. Rod-shaped PEAK achieved higher cellular internalization than spherical counterparts [113,114], correlating with enhanced tumor accumulation and therapeutic outcomes.

Given the instability of bicarbonate under acidic conditions, the pH-triggered morphologically transformable GKRR (β-D-glucopyranoside-KLVFFGGP-RAGLQFPVGR(LLRR)_2_LLR) NPs stabilized by hydrogen bonding between the bicarbonate and guanidine on arginine was fabricated [115]. Tumor acidic microenvironment triggered bicarbonate removal, inducing NPs-to-NFs conversion. The resultant α-helical NFs selectively lysed cancer cells via membrane disruption, overcoming the non-targeting limitations of free peptides.

Based on the pH-responsive property of the insulin-derived oligopeptide VEALYL forming amyloid fibrils under acidic conditions while remaining soluble at neutral pH, the D-LTPS nanotherapeutic was engineered via N-terminal 2-naphthylacetyl conjugation (enhancing self-assembly) and C-terminal glycosylation (improving proteolytic resistance), featuring D-type amino acids throughout its sequence [116]. D-LTPS self-assembled into NPs at pH 7.4 but transformed into NFs in lysosomal acidity (pH 5). This morphological shift induced lysosomal swelling and permeabilization, inducing the necrosis and apoptosis of cancer cells, enabling entrapped chemotherapeutic agents release and overcoming drug resistance. In xenograft drug-resistant tumor models, D-LTPS/chemotherapy combinations achieved superior tumor suppression relative to monotherapy.

**Fig. 8:**
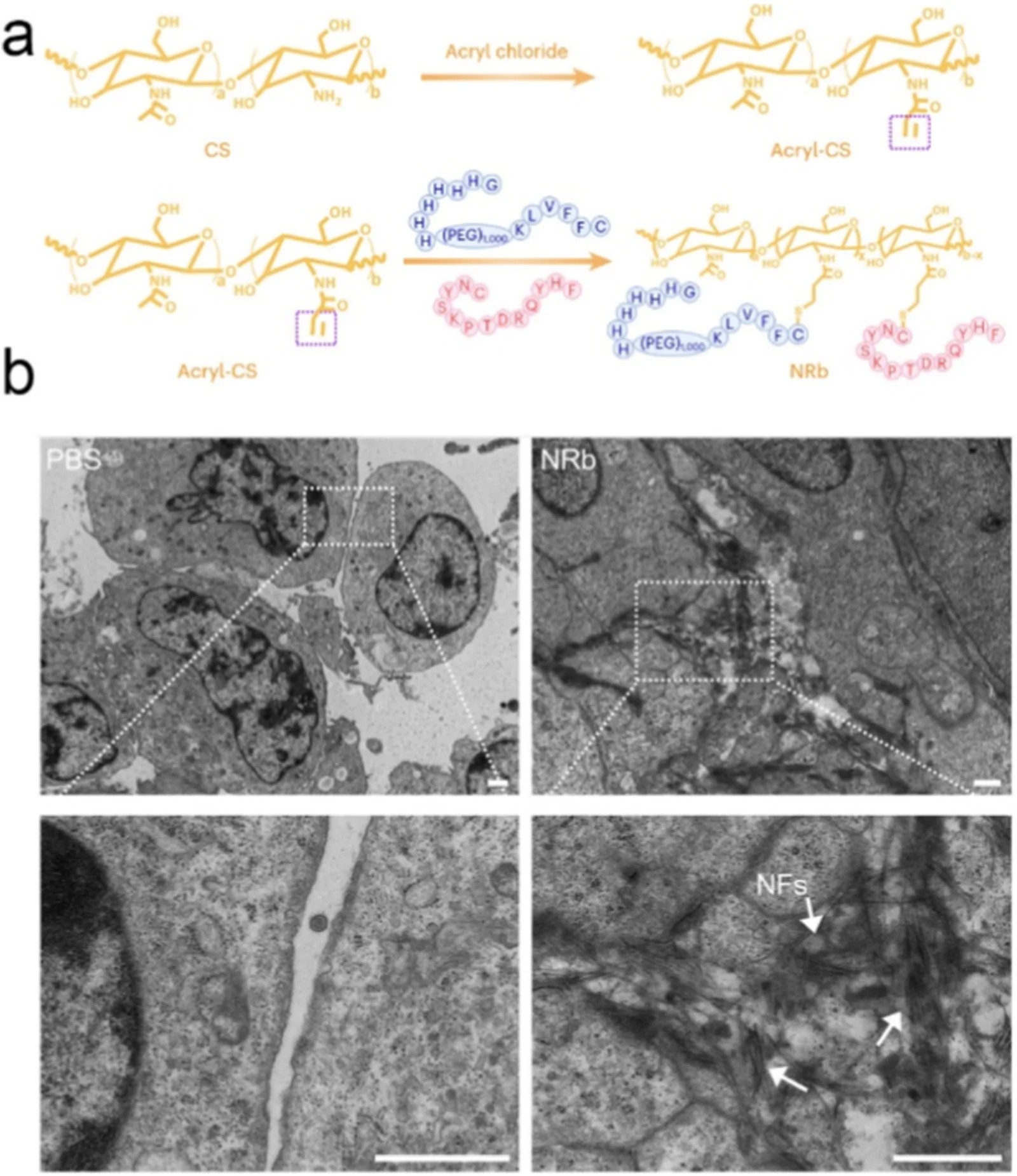
a) The chemical structure of NRb. b) Bio-TEM images of tumor tissues treated without and with NRb NPs. Reproduced with permission: Copyright 2025, Springer Nature [117].

In contrast to above nanotherapeutics, pH-responsive nanotherapeutics exerted antitumor effects through a distinctive membrane-rupturing mechanism. In a recent study, a dual-functional peptide–polymer conjugate, designated NRb, was designed for concurrent membrane disruption and PD-1/PD-L1 blockade [117]. NRb comprised three modules: CNYSKPTDRQYHF (named P) for PD-L1 binding, CFFVLKK-(PEG)_1000_-H_6_G (named K) for pH-responsive morphological transformation, and chitosan as the core framework (Fig. 8a). Upon intravenous administration, NRb NPs accumulated on tumor cell membranes via PD-L1 binding. Simultaneously, histidine protonation triggered by the mildly acidic microenvironment induced in situ transformation into NFs (Fig. 8b). This morphological transformation further enhanced the blockade of PD-1/PD-L1 interactions. Notably, the mechanical force generated during the transformation process resulted in rupture of the cell membrane.

The peptide amphiphile NBD-LLLLpY (NLY), comprising nitrobenzoxadiazole, phosphotyrosine, and a leucine-rich tetrapeptide, selectively targeted ALP-overexpressing osteosarcoma cells [118]. NLY self-assembled into micelles in PBS, with ALP-mediated dephosphorylation inducing a transition to nanoribbons. This structural conversion conferred enhanced cytotoxicity toward osteosarcoma cells, supporting the ALP-triggered micelle-to-nanoribbon transition as the mechanism for selective cancer cell killing. The ALP-based selective cytotoxicity of NLY extended to ALP-overexpressing human induced pluripotent stem cells [119]. ALP-responsive nanotherapeutics can also selectively disrupt cancer cell membranes. The peptide-polymer conjugate PNpC, consisting of poly(vinyl alcohol) (PVA), CM11 peptide and NapFFpYC, accumulated at tumor sites following intravenous administration [120]. ALP-triggered a NPs-to-NFs transition on cancer cell membranes, where NFs enhanced CM11-mediated membrane disruption while reducing cellular internalization. This process promoted the release of damage-associated molecular patterns (DAMPs), which facilitated DC maturation and CD8^+^ T cell infiltration to amplify antitumor immunity.

CtsB is a prominent lysosomal protease that is frequently overexpressed in a variety of malignant tumors [[121], [122], [123]]. Inspired by these findings, NDI-Lyso-RGD was constructed for selective targeting and eradication of tumor cells through lysosomal membrane permeabilization upon activated by CtsB. NDI-Lyso-RGD comprised self-assembling FF peptides, fluorescent hexyl naphthalene diimide alanine (C6NDI-Ala), CtsB-cleavable RRRRK and tumor-targeting RGD [124]. Intravenous administrated NDI-Lyso-RGD NPs accumulated within the lysosomes of cancer cells, where CtsB cleavage induced fibrous elongation, causing lysosome swelling and membrane disruption to trigger apoptosis.

Mitochondria, the primary intracellular source of ROS [125], serve as an ideal subcellular target for selective tumor cell eradication. The peptide-polymer conjugate P1(R1-KLAK) contained a ROS-cleavable thioketal linker within the R1 segment (CGGGKLVFF-tk-PEG) and a mitochondria-targeting cytotoxic peptide (KLAKLAK)_2_ [126]. Upon H_2_O_2_ exposure, thioketal bond cleavage triggered a morphological transition from NPs to NFs, which significantly enhanced tumor accumulation and retention compared to the non-responsive counterpart, thereby augmenting in vivo antitumor activity. Following a similar design strategy, GSH-responsive PKK-ss-PEG NPs underwent PEG shell shedding post-endocytosis and transformed into NFs, exposing (KLAKLAK)_2_ motifs to induce mitochondrial disruption [127]. Another example of mitochondria-targeting NPs was the peptide-porphyrin conjugate TGFK (TCPP-GG^D^F^D^F^D^YCG^D^K^D^R^D^K, D refers to D-amino acids), which integrated a ROS-responsive cysteine residue, a mitochondrial-targeting G^D^K^D^R^D^K sequence, self-assembling backbone GG^D^F^D^F^D^Y and the sonosensitizer porphyrin TCPP, enabling ultrasound-triggered ROS eruption for selective apoptosis induction [30]. ROS-mediated oxidation triggered TGFK to self-assemble into disulfide-bridged dimers, driving a dynamic NPs to NFs transition. Upon cellular internalization, mitochondria-targeting TGFK NPs underwent ROS-triggered crosslinking into NFs with multivalent binding motifs, enhancing mitochondrial adhesion. Under ultrasound irradiation, these NFs generated localized ROS burst, inducing mitochondrial membrane depolarization and subsequent apoptosis.

The integration of phototherapeutic modalities expands the function of peptide-based nanotherapeutics. For instance, Ce6-decorated β-cyclodextrin (Ce6-CD) formed stable NPs with ferrocene-modified FFVLG_3_C-PEG (Fc-FGC-PEG) through the embedding of the hydrophobic Fc moiety into the β-CD cavity [128]. In tumor microenvironments, excess ROS oxidized Fc to hydrophilic Fc^+^, dissociating the inclusion complex. Ce6-CD re-assembled into smaller NPs to enhance deep tumor penetration, while Fc^+^-FGC-PEG formed NFs to prolong retention. Laser irradiation activated Ce6 to generate ^1^O_2_, which synergized with the Fc^+^-mediated Fenton reaction cascade to produce •OH and O_2_, amplifying PDT efficacy. In another example, photosensitizer pheophorbide a (Pa) and LXY30 ligand peptide targeting α_3_β_1_ integrin were connected to the lysine residues at both ends of the self-assembling backbone peptide KLVFFK, resulting the LRI-triggered morphologically transformable nanotherapeutics LKKPa [129]. LKKPa NPs bound cell membrane integrins, transforming into NFs that induce Pa aggregation into J-aggregates. This morphological transition boosted photothermal conversion efficiency and ROS generation compared to non-transformed NPs, enabling a low dosing frequency (once weekly) and low dosage which lowered administration-related injury.

Integrating photosensitizers into peptide assemblies also offers a straightforward strategy for engineering light-triggered nanotherapeutics. The multifunctional peptide amphiphile TRFC (TPP-RRRKLVFFK-Ce6) incorporated Ce6 for ROS generation, KLVFF as the assembly backbone, oligoarginine RRR as a nitric oxide (NO) donor and triphenylphosphonium (TPP) for mitochondrial targeting. This design enabled ROS-mediated arginine oxidation that concomitantly triggered NO release and transformed NPs into nanorods [130]. Released NO was further oxidized to cytotoxic peroxynitrite (ONOO^−^), thereby enhancing PDT efficacy. Irradiation-dependent nanorod formation was observed, which enhanced tumor retention by promoting cellular uptake. In vivo fluorescence imaging demonstrated that the tumor accumulation of irradiated TRFC was approximately 3-fold higher than that of non-irradiated controls. Substituting RRR with KKK to generate TKFC reduced cytotoxicity and tumor-suppressive effects, confirming dual roles of RRR in NO release and PDT efficacy amplification. This spatiotemporally controlled nanotherapeutics exemplifies how dynamic nanoscale morphological reconfiguration can enhance synergistic therapeutic efficacy.

#### 4.2.2 Nanotherapeutics for bacterial infection

**Fig. 9:**
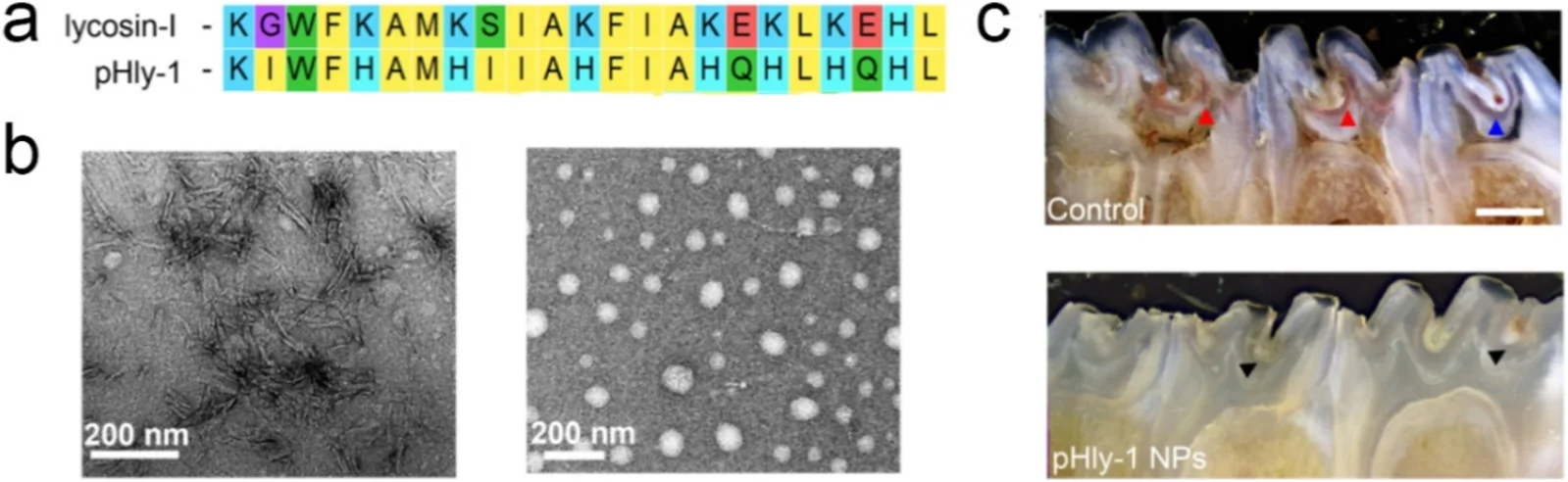
a) The peptide sequence of lycosin-l and pHly-1. b) TEM images of the pHly-1 at pH 7.0 (left) and 4.5 (right). c) Representative photos of carious lesions without and with pHly-1 treatment. Black, blue, and red arrows indicate initial, moderate, and extensive lesions, respectively. Reproduced from Ref. [136], licensed under CC BY 4.0. (For interpretation of the references to colour in this figure legend, the reader is referred to the Web version of this article.)

Bacteria in biofilms show 10- to 1000-fold greater antibiotic resistance than unprotected counterparts [131,132]. Their anaerobic metabolism generates an acidic microenvironment (pH 4.5–6.5) within biofilms [133,134]. Antimicrobial peptides (AMPs) disrupt bacterial membranes for antimicrobial activity but suffer from off-target cytotoxicity. To address this, pH-triggered transformable nanotherapeutics leverage infection-site acidity for selective bactericidal effects. Covalent modification of lysine side chains of C_16_-A_3_K_4_-CONH_2_ with DMA yielded C_16_-A_3_K_4_(DMA)-CONH_2_, which self-assembled into negatively charged NPs [135]. Acidic conditions cleaved DMA, triggering NP conversion to cationic nanorods. These nanorods bound bacterial membranes more strongly via increased contact area, effectively inhibiting in vivo infections. As a complementary strategy, pHly-1, engineered by site-specific amino acid substitution of lycosin-I (Fig. 9a), formed biocompatible NFs at neutral pH. In cariogenic biofilms (pH < 6.5), NFs switched to membrane-lytic NPs (Fig. 9b) to reduce dental caries [136]. In vivo studies showed rats treated with pHly-1 had significantly fewer carious lesions than untreated controls (Fig. 9c), confirming site-specific antimicrobial efficacy.

For biofilm-penetrating therapy, CHFK(PEG)-QT integrated four functional modules, including 14-carbon alkyl chain for providing hydrophobic forces, peptide sequence (HHHF)_4_HHHK as pH-responsive and β-sheet-forming motif, hydrophilic PEG chain for improved biocompatibility and assembly stability and QRKLAAKLT for targeting *P. aeruginosa* [137]. At biofilm acidity (pH 5.0), histidine protonation triggered NFs-to-NPs transitions, exposing cationic surfaces that disrupted bacterial membranes more effectively than assemblies at neutral pH. This microenvironment-specific activation overcame biofilm-mediated antibiotic resistance while maintaining systemic safety in porcine infection models.

Exploiting gelatinase specifically secreted by staphylococcus aureus (*S. aureus*) [138], the CPC-1 nanotherapeutic consist of a chitosan backbone, antimicrobial CGGG(KLAKLAK)_2_, and gelatinase-cleavable PEG_2000_GPLGVRGC was designed to target infectious sites [139]. Circulating NPs maintain stability via PEG shielding. At infection foci, gelatinase-mediated PEG removal induced NPs-to-NFs transformation, exposing AMPs to enhance bacterial membrane affinity and bactericidal efficacy. Morphologically persistent control CPC-2 (GPMGMRGC replacing GPLGVRGC) showed reduced retention in *S. aureus*-infected mice compared to CPC-1, validating the advantage of enzyme-triggered morphological transformation.

**Fig. 10:**
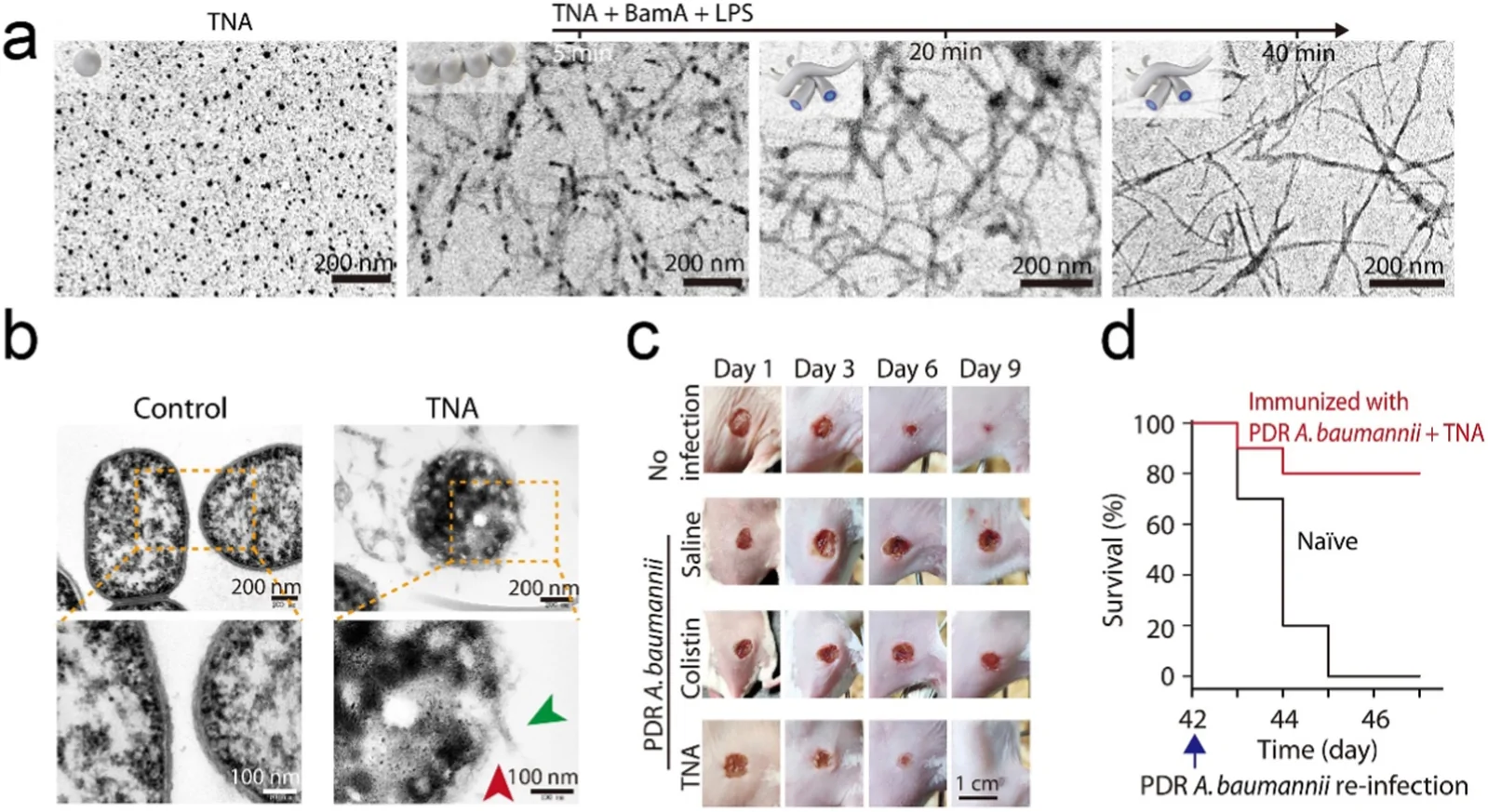
a) TEM images of TNA before and after incubation with BamA and LPS. b) bio-TEM images of untreated (control) and TNA-treated PDR *A. baumannii*. c) Representative photographs of the infected area of the PDR *A. baumannii*–infected mice in different groups. d) Survival of mice intra-abdominal rechallenged with PDR *A. baumannii* in different groups. Reproduced with permission: Copyright 2023, AAAS [140].

For combating drug-resistant Gram-negative bacteria, TNA was designed to target outer membrane LPS and β-barrel assembly machine A (BamA) [140]. TNA composed of a pyrene (Py) core, β-sheet motif FF, LPS-targeting KKK, and BamA-recognizing sulfanilamide. Binding to LPS and BamA triggered the transformation from NPs to NFs (Fig. 10a). The rigid NFs mechanically disrupted bacterial outer membranes (Fig. 10b), inducing the death of bacteria and releasing antigens to activate adaptive immunity. TNA exhibited potent in vivo bactericidal effects and durable protective activity as demonstrated in wound (Fig. 10c) and peritonitis (Fig. 10d) models involving infection with drug-resistant *A. baumannii*.

Unlike membrane-rupturing nanotherapeutics, some strategies fight bacteria by activating host immunity. Sepsis-induced immunosuppression causes secondary infections, and the peptide nanotherapeutic BATMAN5 was developed to restore macrophage antibacterial activity [141]. Composed of four functional modules, targeting UBI29-41, lipase-sensitive group, self-assembling FFVLK, and immunostimulatory tuftsin peptide, BATMAN5 NPs shield tuftsin initially. Following intravenous administration, the NPs preferentially accumulated on bacterial surfaces via the targeting peptide. Elevated bacterial lipase levels in the infectious microenvironment triggered the transformation into NFs, exposing clustered tuftsin. This enhanced macrophage Fcγ receptor binding, promoted bacterial phagocytosis, and drived macrophage repolarization to an antibacterial phenotype.

#### 4.2.3 Nanotherapeutics for other diseases

**Fig. 11:**
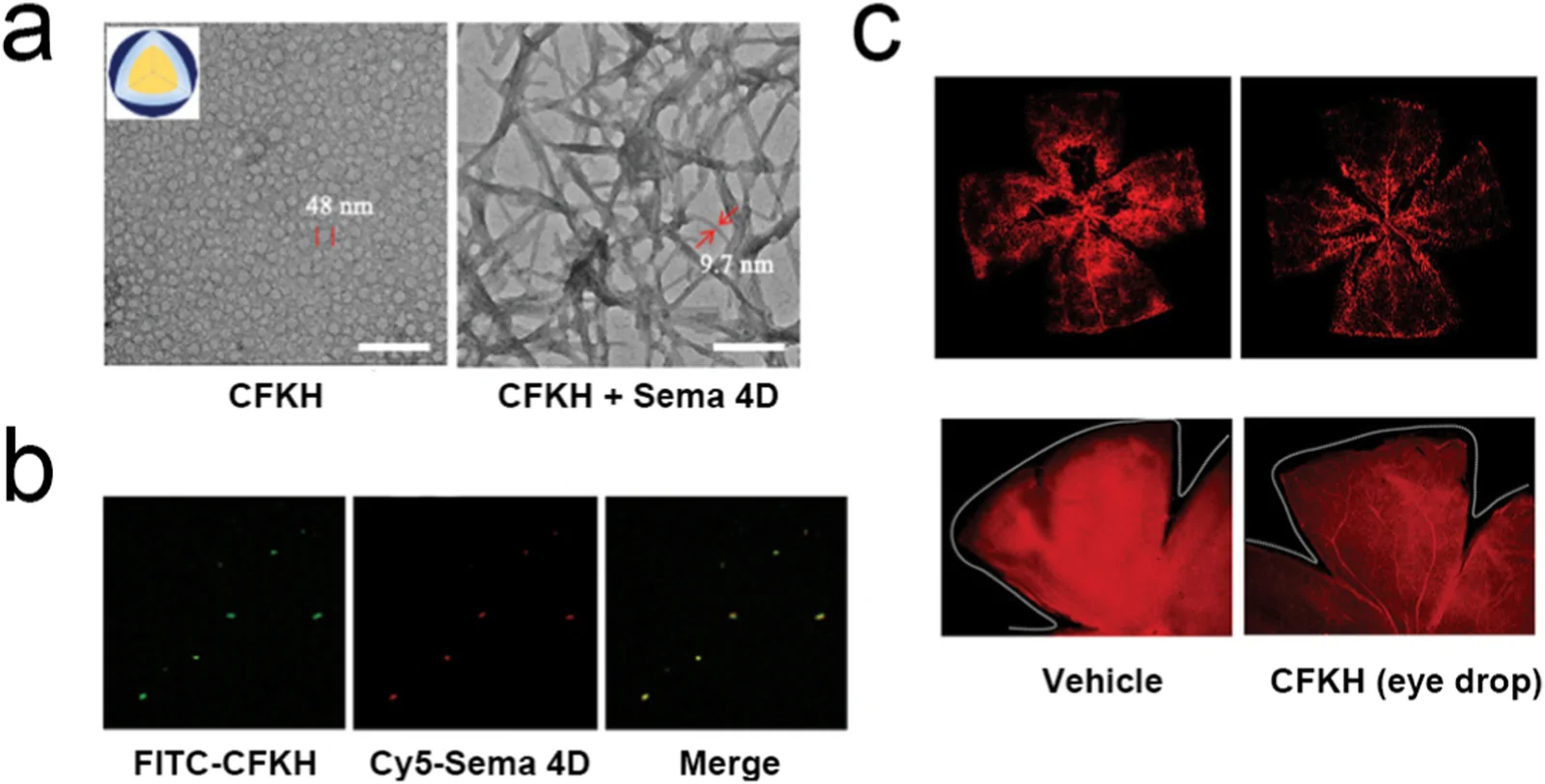
a) TEM images of CFKH before and after incubation with Sema 4D. b) CLSM images showing specific recognition and capture of Sema 4D (red) by CFKH (green). c) CFKH significantly reduced abnormal neovascularization in an oxygen-induced retinopathy mouse model (top) and retinal vascular leakage in a diabetic mouse model (bottom). Reproduced from Ref. [142], licensed under CC BY 4.0. (For interpretation of the references to colour in this figure legend, the reader is referred to the Web version of this article.)

LRI-triggered nanotherapeutics have emerged for treating inflammatory diseases, leveraging specific molecular recognition to modulate pathogenic mediators. A representative example is CFKH nanotherapeutics, designed based on palmitoleic acid, FFVLK, and soluble semaphorin 4D (sSema4D)-targeting peptide KQKRPRH. sSema4D is a pro-angiogenic factor overexpressed in diabetic retinopathy (DR). The nanotherapeutics transform from NPs to fibrous networks (Fig. 11a) and sequester sSema4D (Fig. 11b) [142]. In oxygen-induced retinopathy and diabetic mouse models, CFKH nanotherapeutics markedly reduced retinal neovascularization and vascular leakage (Fig. 11c). Following the same LRI design principle, BP-KF-SG/HL NPs targeted IgE. IgE is a key mediator of allergic rhinitis. The two peptide amphiphiles consists of functional peptides SILPVDAKDWIEGEG or HPEYAVSVLL conjugated to KLVFF and BP, respectively. They transformed into nasal mucosal fibrillar networks upon IgE binding to trap IgE and block mast cell activation [143]. Murine models demonstrated reduced inflammatory symptoms and cytokine levels, highlighting the potential of LRI-triggered nanotherapeutics in allergic disease management.

### 4.3 Biomimetic nanotherapeutics

Inspired by natural biological systems, biomimetic strategies further optimize the therapeutic performance of morphologically transformable peptide nanomaterials by mimicking endogenous structures and functions. Nature has evolved diverse biological materials with sophisticated functionalities [144], inspiring innovative biomedical advances [[145], [146], [147]]. Modern biomedicine leverages endogenous substances and biological principles to develop novel therapies, exemplified by therapeutic antibodies [148], cellular therapies [149], and gene therapy. Biomimetic approaches enable the rational design of advanced therapeutics that transcend mere replication of biological counterparts, incorporating morphological and functional optimizations for enhanced performance. Particularly, supramolecular self-assembly of bioactive peptides emerges as a powerful paradigm for developing dynamically reconfigurable nanotherapeutics. Unlike conventional nanotherapeutics (mentioned above), which are designed to target specific pathological entities (e.g., aberrantly expressed enzymes or proteins) and act through well-defined mechanisms such as target blockade or direct pathogen killing, biomimetic nanotherapeutics mimic endogenous functional components and exert therapeutic effects primarily by leveraging the biological functions they emulate. This section highlights biomimetic morphologically transformable peptide-based nanotherapeutics for disease treatment, emphasizing their therapeutic advantages through programmable morphological transformation.

#### 4.3.1 ECM/platelet-mimetic nanotherapeutics for cancer

ECM remodeling is a key driver of tumor progression and metastasis [150]. An approach has been developed by engineering tumor-localized ECM-mimetic supramolecular networks [151]. The laminin-inspired peptide amphiphile BKGY (BP-KLVFFK(GGDGR)YIGSR) integrated fluorescent BP, β-sheet-forming KLVFF, and dual-receptor-targeting RGD/YIGSR motifs. Intravenously administered BKGY NPs utilize the EPR effect and tumor-specific LRI to accumulate at tumor sites, where they form nanofibrillar matrices. These matrices resisted degradation by tumor-derived proteases and physically block tumor cell dissemination and invasion. In murine models of breast and melanoma lung metastasis, BKGY NPs significantly reduced metastatic nodule formation compared to control groups, attributed to the ECM-like physical barrier. Complementing this laminin-inspired strategy, a fibronectin-mimetic peptide (FNMP) was engineered with integrin-responsive fibrillogenesis activity [152]. The FNMP formulation (BP-K-FFVLK-CREKA) leveraged integrin-mediated recognition to trigger on-site self-assembly into proteolytically resistant fibrillar barriers on integrin-overexpressing tumor cells. This synthetic framework retained fibronectin-mimetic bioactivity while overcoming the inherent proteolytic vulnerability of natural fibronectin, simultaneously blocking invasion-related receptors on tumor cells. In vivo studies confirmed that FNMP suppresses tumor invasion and distant metastasis effectively. Together, these studies validated pathology-triggered morphological remodeling as a robust strategy, wherein supramolecular nanomaterials transition from inert carriers to bioactive ECM mimics. This approach enables spatiotemporal control of the tumor metastatic microenvironment via biomimetic topological remodeling, offering a promising avenue for anti-metastatic therapy.

Expanding this concept, pH-responsive LMP-2 NPs composed of the KLVFF motif, microthrombus-targeting CREKA sequence, and pH-sensitive His_6_ motif, were constructed to mimic laminin fibrillogenesis in tumor vasculature [153]. The acidic tumor microenvironment induces the NPs-to-NFs transition, with the thrombus-targeting CREKA further accelerating this morphological rearrangement. This dual-functional platform exhibited prolonged tumor retention compared to non-transformable NPs, thereby inhibiting tumor progression through vascular occlusion. Using platelet activation biomimicry, CD105-targeted BFA NPs (BP-FFVLK-AHKHVHHVPVRL) were developed for tumor vascular occlusion [154]. These platelet-mimetic BFA NPs specifically targeted CD105-overexpressing tumor vasculature, forming artificial fibrin clots through ligand-induced NFs crosslinking. This artificial thrombosis disrupted tumor blood supply, leading to marked inhibition of tumor growth in murine models.

#### 4.3.2 Antibody-mimetic nanotherapeutics for corneal neovascularization

One of the mechanisms of action of antibodies involves blocking receptors or ligands critical to disease progression [155]. Traditional molecular-format antibodies are primarily proteins, which are typically expensive to produce, complex, and unstable. Morphologically transformable antibody-mimetic peptide nanotherapeutics serve as a cost-effective, stable substitute for conventional protein-based antibodies by leveraging dynamic shape-shifting to disrupt LRIs. BKP (BP-KLVFF-PCAIWF) NPs transformed into NFs upon binding to VEGFR, blocking downstream signaling pathways and thereby inhibiting angiogenesis [156]. The non-transformable variant C-BKP (with GGAAK substitution) failed to form NFs, underscoring the necessity of KLVFF-mediated assembly. Similarly, integrin αvβ3-targeted PA-F(G)H (palmitic acid-FFVLK(GSG)-HSDVHK) underwent NFs transformation on activated endothelial cells, suppressing corneal neovascularization (CNV) through dual anti-angiogenic and anti-inflammatory activities [157]. Notably, this nanotherapeutic outperformed bevacizumab in vivo.

#### 4.3.3 HD6-mimetic nanotherapeutics for bacterial infection

**Fig. 12:**
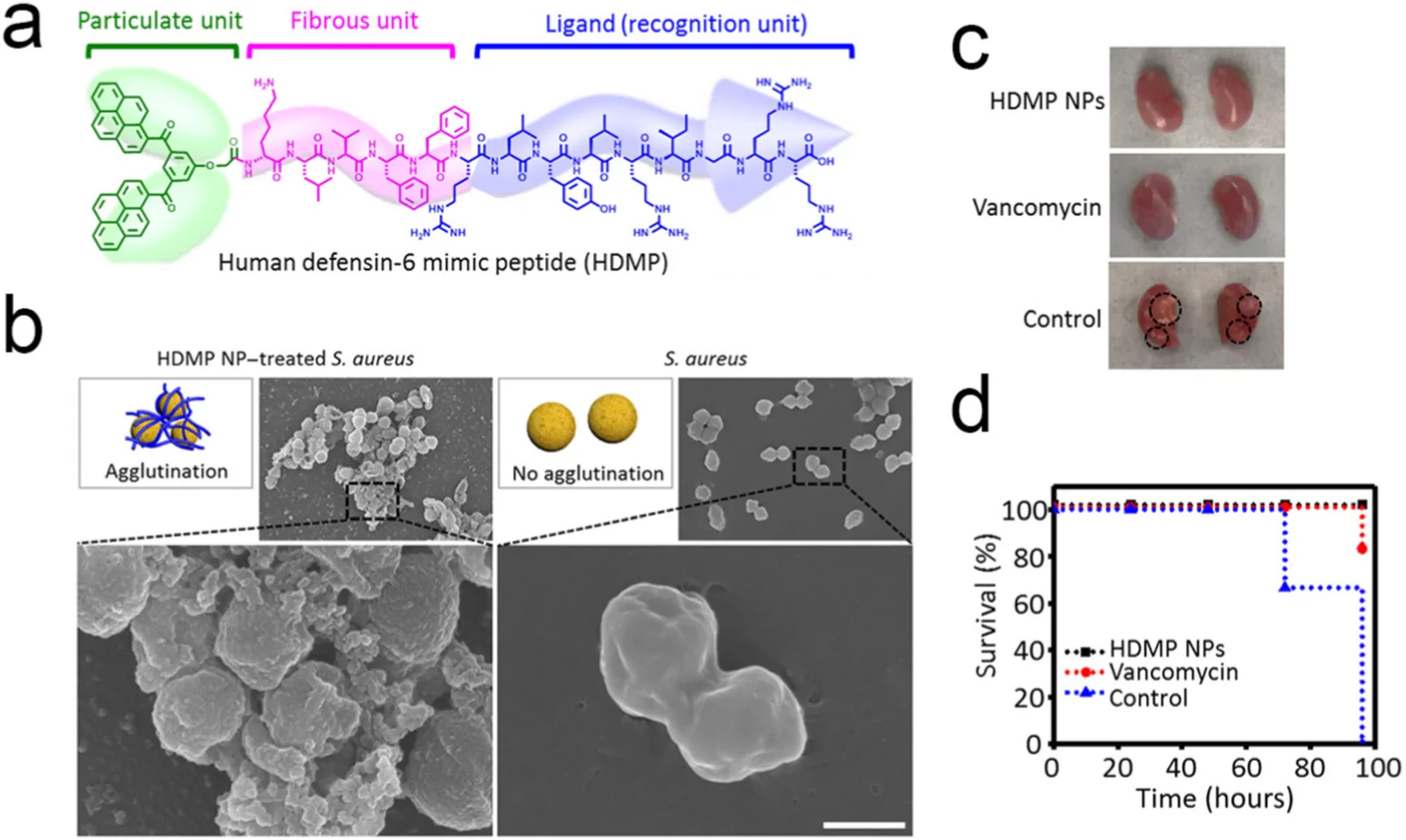
a) The chemical structure of HDMP. b) Schematic and SEM images of *S. aureus* treated with or without HDMP NPs. c) Representative images of MRSA-infected kidneys in different groups. d) Survival curves of bacteremic mice infected with MRSA in different groups. Reproduced with permission: Copyright 2020, AAAS [158].

Inspired by human defensin-6 (HD6), HDMP NPs, composed of RLYLRIGRR targeting lipoteichoic acid (LTA), KLVFF sequence, and hydrophobic BP were designed (Fig. 12a) [158]. HDMP selectively bound Gram-positive bacteria via LTA, forming β-sheet fibrillar networks that entrapped pathogens (Fig. 12b). Highly effective in vivo antibacterial effect of HDMP NPs was verified by mouse models of bacteremia with methicillin-resistant *Staphylococcus aureus* (MRSA) (Fig. 12c). Importantly, survival rate of infected mice treated by HDMP NPs were significantly higher than that treated with vancomycin (Fig. 12d).

## 5 Conclusion and perspective

In summary, recent advances of smart morphologically transformable peptide nanomaterials for drug delivery and as nanotherapeutics are discussed. As an important class of building blocks, peptides possess a wide range of excellent properties, such as bioactivity, biocompatibility, easiness for chemical modification. Additionally, more functional peptide-based building blocks can be constructed by introducing other functional molecules, such as polymers, small molecule drugs, and antibodies. The supramolecular peptide nanomaterials formed by self-assembly are stabilized by weak intermolecular interactions, which endow the assemblies with morphological transformability. Different from the healthy physiological microenvironment, lesions in various diseases exhibit unique microenvironments characterized by abnormal pH, high redox levels, or overexpression of certain enzymes or receptors and so forth, which are regarded as stimuli for responsive nanomaterials design. These stimuli change the forces including noncovalent and covalent interactions, driving the morphological transformation. By integrating stimuli responsiveness and morphological tunability, morphologically transformable peptide nanomaterials can be fabricated for drug delivery and as therapeutics by reasonable molecular design. The representative smart peptide-based nanocarriers (Table 1) and nanotherapeutics (Table 2) along with their molecular types, responsive stimuli, morphological transformations, applications, advantages or mechanism are summarized, respectively.

**Table 1:** Summary of representative morphologically transformable peptide nanomaterials for drug delivery from five aspects including the molecular type, stimulus, morphology transformation, application, and advantage.

Nanocarriers	Molecular type	Stimulus	Morphology transformation	Application and advantage
BP-FFVLK(PEG)-H_6_ [60]	peptide-polymer conjugate	pH	NPs to NFs	cancer, enhanced local retention
DPC-PL [61]	peptide-drug conjugate and polymer	pH	NPs to NFs	cancer, enhanced targeted accumulation
D-ss-P@AR-PL [62]	peptide-drug conjugate and polymer	pH	NPs to spherical network	cancer, enhanced targeted accumulation
ACI [63]	peptide-drug conjugate	pH	NFs to NPs	cancer, enhanced deep penetration and targeted accumulation
AmpFY9 [64]	peptide-drug conjugate	pH	NFs to NPs	cancer, enhanced deep penetration and targeted accumulation
SUN-NPA [65]	peptide-drug conjugate	pH	NFs to NPs	cancer, enhanced targeted accumulation
CPT-FLPR [66]	peptide-drug conjugate	enzyme, CtsB	NPs to NFs	cancer, achieve specific and long-term retention
Nap-FF-K(AZD8055)pY [67]	peptide-drug conjugate	enzyme, ALP	NPs to NFs	cancer, selectively sensitizing tumors
SgVEIP [70]	peptide amphiphiles	enzyme, β-galactosidase	short bundled NFs to long NFs	cancer, enhanced nuclear targeted delivery
AmpFQB@Dex [81]	peptide	enzyme, NQO1	NPs to NFs	acute lung injury, enhanced retention at inflammation sites
MRP@Dox [83]	peptide and peptide amphiphiles	enzyme, legumain	NPs to NFs	fundus neovascularization, enhanced ocular surface penetration and fundus retention
KPGF@bFGF [84]	peptide	enzyme, MMP3	NPs to NFs	MI, enhanced long retention
CPC [59]	peptide-drug conjugate and cucurbit [7]uril	polyamine	NPs to microfibers	cancer, enhanced targeting accumulation and retention
Ce6/BR-FFVLK-PEG [72]	peptide-polymer-drug conjugate	laser	NPs to NFs	cancer, enhanced retention and deep penetration
Dox@C12-KF-NL [74]	lipopeptide	LRI	NPs to nanofibrous coating	cancer, enhanced retention
NCefoTs [82]	lipopeptide	protein, DsbA	NPs to NFs	drug-resistant infections, enhanced bacterial outer membrane penetration
MEL/Cypate@HA [79]	peptide and polymer	pH and laser	NPs to NFs to NPs	cancer, enhanced retention and deep penetration
FASP [76]	peptide-drug conjugate and polymer	GSH and esterase	NPs to NFs	cancer, enhanced cellular uptake and radiosensitization
PEG-His@BPC [77]	peptide-drug conjugate and polymer	pH and MMP2	NPs to NFs	cancer, enhanced intratumoral retention
HpYss [78]	peptide-drug conjugate	GSH and ALP	NPs to NFs	cancer, enhanced nuclear targeted delivery
Nap−CPT−HCQ−pY [80]	peptide-drug conjugate	enzyme, ALP, CES	nanobrush−NPs−NFs	cancer, enhanced cellular uptake and retention

**Table 2:** Summary of representative morphologically transformable peptide nanotherapeutics from five aspects including molecular type, stimulus, morphology transformation, application, and mechanism.

Nanotherapeutics	Molecular type	Stimulus	Morphology transformation	Application and mechanism
PWG [111]	peptide amphiphiles	pH	NPs to NFs	cancer, in situ forming NFs for enhanced PDT
PEAK [112]	peptide amphiphiles	pH	NPs to nanorods	cancer, in situ forming nanorods for enhanced PDT
GKRR [115]	peptide amphiphiles	pH	NPs to NFs	cancer, transformed into therapeutic NFs from non-toxic NPs
D-LTPS [116]	peptide amphiphiles	pH	NPs to NFs	cancer, initiating lysosomal hydrogelation for swelling lysosome
CHFK(PEG)-QT [137]	peptide amphiphiles	pH	NFs to NPs	drug-resistant bacteria infection, transformed into therapeutic NPs from non-toxic NFs
TEP-FFG-CRApY [87]	peptide amphiphiles	enzyme, ALP	NPs to NFs	cancer, triggering immunogenic ferroptosis through combination of PDT and LMP
PAC-SABI [100]	peptide-ploymer and protein	enzyme, ALP	NPs to NFs	cancer, in situ transformed into NFs for blocking CD47 and CD24 signaling
NLY [118]	peptide amphiphiles	enzyme, ALP	NPs to nanoribbons	osteosarcoma cell, binding with histone of nuclei via morphological transformation
PAC [89]	peptide amphiphiles	enzyme, O-GlcNAcase	NPs to NFs	cancer, long-term retention to stimulate PKM2 tetramerization and recover chemotherapeutic sensitivity
EIsYBE3 [92]	peptide amphiphiles	enzyme, sulfatase	NPs to NFs	cancer, degradation of Bcl-xL and activation of caspase-dependent apoptosis
NDI-Lyso-RGD [124]	peptide amphiphiles	enzyme, CtsB	NPs to NFs	cancer, induce lysosomal swelling and trigger cancer cell apoptosis
CPC-1 [139]	peptide-ploymer conjugate	enzyme, gelatinase	NPs to NFs	bacterial infection, transformed to membrane-rupturing NFs from nontoxic NPs
BP-FFVLK-RLPS [97]	peptide amphiphiles	LRI	NPs to NFs	cancer, target and in situ stimulating microtubules condensation
BFYR [98]	peptide amphiphiles	LRI	NPs to NFs	cancer, target and in situ stimulating microtubules condensation
TPM [104]	peptide amphiphiles	LRI	NPs to NFs	cancer, in situ forming NFs to disrupt HER2 dimerization
LKKPa [129]	peptide amphiphiles	LRI	NPs to NFs	cancer, in situ forming network around cells surfaces for enhanced phototherapy
TNA [140]	peptide amphiphiles	LRI	NPs to NFs	antibiotic-resistant bacterial infection, in situ transforming rigid NFs to damage the bacterial envelope
CFKH [142]	peptide amphiphiles	LRI	NPs to NFs	diabetic retinopathy, recognize and capture soluble semaphorin4D
BKGY [151]	peptide amphiphiles	LRI	NPs to NFs	cancer, in situ forming network to inhibit tumor metastasis
FNMP [152]	peptide amphiphiles	LRI	NPs to NFs	cancer, in situ forming network to inhibit tumor cells migration and invasion
LMP-2 [153]	peptide amphiphiles	LRI	NPs to NFs	cancer, in situ forming fibrous network as artificial clots to inhibit tumor growth
BFA [154]	peptide amphiphiles	LRI	NPs to NFs	cancer, in situ form artificial blood clots for inhibiting tumor growth upon binding CD105
HDMP [158]	peptide amphiphiles	LRI	NPs to NFs	antibiotic-resistant bacterial infection, in situ forming nanofibrillar network and trapping bacteria
TGFK [30]	peptide amphiphiles	ROS	NPs to NFs	cancer, transformed into NFs for sonodynamic therapy from nontoxic NPs
P1(R1-KLAK) [126]	peptide amphiphiles	ROS	NPs to NFs	cancer, transformed into mitochondrial-destroying NFs from nontoxic NPs
TRFC [130]	peptide amphiphiles	ROS	NPs to nanorods	cancer, enhance PDT for selectively killing tumor cells

Compared with drug delivery nanocarriers with unchanged morphology, morphologically transformable peptide nanomaterials for drug delivery exhibit different morphologies at different delivery stages, which is more conducive to overcoming multiple biological barriers and thus improving enrichment and penetration of drugs at target tissues. Likewise, morphologically transformable peptide-based nanotherapeutics exhibit a superior performance in overcoming multiple biological barriers than morphologically persistent counterpart. These peptide-based nanotherapeutics treat disease through a variety of therapeutic mechanisms, including blocking important proteins or receptor-mediated biological functions through morphological transformation, and transformation from non-toxic nanostructures to nanostructures with killing properties (mainly for anticancer and antimicrobial therapy). In addition, peptide-based nanotherapeutics possess significant scalability and can be further integrated with advanced therapeutic modalities, such as PROTACs. Here, we summarize some common molecular characteristics of these peptide-based building units: i) in terms of physicochemical properties, they are basically amphiphilic; ii) they are usually composed of several functional motifs, among which stimuli-responsive and targeting motifs are necessary. As a multifaceted player, the peptide can serve as a targeting motif, a responsive motif, or a self-assembling core motif in the systems. In general, the design of these drug delivery nanocarriers and nanotherapeutics mainly follows modular design principles. Similar to Lego bricks, it enables the construction of tailored entities from a selection of functional modules to meet specific requirements.

Even though smart morphologically transformable peptide-based drug nanocarriers and nanotherapeutics have shown promising therapeutic potential for disease treatment, there are some key issues that need to be addressed. While for nanomaterials that respond to pH, enzymatic, and redox stimuli, the mechanism of transformation is relatively clear, i.e., changes in molecular structure disrupts the original hydrophilic-hydrophobic equilibrium, which subsequently induces a change in intermolecular interactions, resulting in the formation of an alternative, thermodynamically more stable nanostructures. The exact mechanism of morphological transformation of LRI-triggered morphologically transformable nanotherapeutics remains largely unclear. Most studies on morphologically transformable peptide nanomaterials have focused on antitumor and antibacterial treatments. Applications in more diseases, such as neurological diseases, metabolic disorders, and autoimmune diseases, remain underexplored and require further investigation. Due to current technological limitations, monitoring the dynamic morphological transformation of nanomaterials in vivo is a major challenge. This lack of direct evidence hinders further clinical translation. Nevertheless, it is believed that these challenges will be gradually overcome with the development of in vivo imaging technology. The design and construction of novel smart morphologically transformable peptide nanomaterials can be accelerated by artificial intelligence technology [159,160] and innovative therapeutic technologies, while these smart peptide nanomaterials are also expected to inspire the design and development of smart drug delivery nanocarriers and nanotherapeutics based on other functional materials.

## CRediT authorship contribution statement

**Jikang Liu:** Writing – original draft, Visualization, Investigation. **Xunan Kan:** Visualization, Investigation. **Yong Su:** Investigation. **Yao Yao:** Visualization. **Jiawen Zhao:** Visualization. **Jianwei Bao:** Writing – original draft. **Yong Jin:** Writing – review & editing, Conceptualization. **Qianli Zou:** Writing – review & editing, Project administration, Funding acquisition, Conceptualization.

## Declaration of competing interest

The authors declare that they have no known competing financial interests or personal relationships that could have appeared to influence the work reported in this paper.

## Data Availability

No data was used for the research described in the article.
